# Structure and assembly of Borna disease virus 1 nucleoprotein-RNA complexes

**DOI:** 10.1126/sciadv.aeb0835

**Published:** 2026-04-10

**Authors:** Yukihiko Sugita, Yuya Hirai, Shinya H. Goto, Takuro Fujiwara, Keizo Tomonaga, Takeshi Noda, Masayuki Horie

**Affiliations:** ^1^ Hakubi Center for Advanced Research, Kyoto University, Kyoto, Japan.; ^2^ Laboratory of Ultrastructural Virology, Institute for Life and Medical Sciences, Kyoto University, Kyoto, Japan.; ^3^ Laboratory of Ultrastructural Virology, Graduate School of Biostudies, Kyoto University, Kyoto, Japan.; ^4^ Department of Biology, Osaka Dental University, Hirakata, Japan.; ^5^ Laboratory of RNA Viruses, Department of Virus Research, Institute for Life and Medical Sciences, Kyoto University, Kyoto, Japan.; ^6^ Department of Molecular Virology, Graduate School of Medicine, Kyoto University, Kyoto, Japan.; ^7^ Laboratory of RNA Viruses, Department of Mammalian Regulatory Network, Graduate School of Biostudies, Kyoto University, Kyoto, Japan.; ^8^ Institute for Integrated Cell-Material Sciences, Kyoto University, Kyoto, Japan.; ^9^ Laboratory of Veterinary Microbiology, Graduate School of Veterinary Science, Osaka Metropolitan University, Izumisano, Japan.; ^10^ Osaka International Research Center for Infectious Diseases, Osaka Metropolitan University, Osaka, Japan.

## Abstract

Structures of nucleoprotein (N)–RNA complexes of the *Bornaviridae*, a virus family in the order *Mononegavirales*, have not been reported. Here, using cryo–electron microscopy (cryo-EM), we report high-resolution structures of Borna disease virus 1 (BoDV-1) N-RNA complex assemblies, including a dominant hexameric ring-like complex and less populated heptameric and octameric forms, the first RNA-bound N structures reported from this family. These structures reveal key features of N-RNA engagement and a BoDV-1–specific stoichiometry of eight nucleotides per N, providing a framework for comparison with related negative-strand RNA viruses. In addition to these RNA-bound complexes, we identified multiple RNA-free oligomers, indicating substantial conformational flexibility of N. Mutational analyses identified residues essential for nucleocapsid formation and RNA synthesis. Cryo-EM of mutant complexes captured RNA-free assemblies, suggesting that initial N oligomerization precedes RNA binding. These findings clarify the structural organization of the N-RNA complex and suggest how oligomeric plasticity contributes to nucleocapsid assembly.

## Teaser

Cryo-EM reveals detailed structures of RNA-bound Borna disease virus 1 nucleoprotein and its RNA-free assemblies.

## INTRODUCTION

Viruses in the order *Mononegavirales* typically have linear, nonsegmented, negative-strand, single-stranded RNA genomes. They infect a wide range of host species in the kingdoms Animalia, Plantae, and Fungi (*1*). Mononegaviruses infecting humans are classified into five families: *Filoviridae* [Ebola (EBOV) and Marburg viruses], *Paramyxoviridae* [measles (MV) and Nipah viruses], *Rhabdoviridae* (rabies virus), *Pneumoviridae* [human respiratory syncytial virus (HRSV)], and *Bornaviridae*, represented by Borna disease virus 1 (BoDV-1). RNA genomes of these viruses are encapsidated by multiple copies of the viral nucleoprotein (N) molecule that forms N-RNA complexes. N-RNA complexes are scaffolds for binding to the viral phosphoprotein (P) and RNA-dependent RNA polymerase (L) and for assembling into functional viral nucleocapsids responsible for viral RNA transcription and replication. In infected cells, biomolecular condensates known as viral inclusion bodies (IBs) are formed as membraneless compartments in which nucleocapsid assembly and viral RNA synthesis are spatially coordinated (*2*–*5*).

Given the essential roles of nucleocapsid architecture in viral replication, extensive structural studies have been conducted on mononegavirus N molecules. A previous x-ray crystallography study provided the first high-resolution structure of a negative-strand RNA virus N by resolving BoDV-1 N in an RNA-free, planar tetrameric complex (*6*). This structure exhibited a bilobed α-helical fold, composed of N- and C-terminal lobes connected by a linker and flanked by extended N- and C-terminal arm domains (fig. S1A). Structural studies of other mononegavirus families have confirmed the conservation of this bilobed core and extended-arm motif (*7*–*17*), with RNA binding consistently occurring in a “cleft” between the lobes, as revealed by multiple N-RNA complex structures (*7*, *8*, *10*, *11*, *16*, *18*). Furthermore, cryo–electron microscopy (cryo-EM) studies of various species have demonstrated structural conservation, particularly at the individual protein level in the same virus families (*19*–*22*), and revealed diverse, oligomeric arrangements of N complexes in both RNA-bound and RNA-free states (*23*–*25*).

BoDV-1, the type species of the family *Bornaviridae*, is a neurotropic pathogen of considerable public health concern, causing fatal encephalitis in various mammals, including humans (*26*). Unlike other human mononegaviruses that replicate in the cytoplasm, BoDV-1 replicates in the nucleus of host cells, where the nucleocapsid associates with host chromatin, facilitating persistent infection through successive host cell divisions (*27*). Given its public health relevance and viral life cycle, structural characterization of BoDV-1 N is particularly important. Discovering structural variability, even in RNA-free states, provides critical insights into N complex dynamics, which are essential for understanding nucleocapsid assembly, genome encapsidation, and the unique nuclear replication strategy used by BoDV-1. However, structural knowledge remains limited to a single RNA-free crystal structure for the family *Bornaviridae* (*6*). Among the five families in *Mononegavirales* that include human-infecting viruses, *Bornaviridae* is the only one for which the N-RNA complex structure has not been characterized, representing a critical and long-standing gap in our understanding of the structural properties of viral N molecules. This gap has hindered comparative and evolutionary understanding of RNA encapsidation principles among mononegavirus families.

In addition, although previous N-RNA complex structures revealed overall architectures, they offered limited insight into how N oligomerizes and binds RNA during encapsidation. It remains unclear whether RNA is required to initiate the assembly process or whether N can form modular preassembled complexes that subsequently engage RNA.

To address this question, we used single-particle cryo-EM to reveal high-resolution structures of BoDV-1 N complex in diverse RNA-free and RNA-bound oligomeric states. These analyses captured a broad range of assembly states and conformations, including unreported configurations such as N-terminally truncated subunits. Structure-guided mutational studies, including characterization of key residues, such as Lys^164^, have identified the RNA binding mechanism and the fundamental structural principle required for IB formation and viral RNA synthesis in host cells. These findings offer critical molecular details about assembly mechanisms of BoDV-1 N, contributing to our understanding of mononegavirus RNA encapsidation strategies and molecular evolution. Moreover, high-resolution structures obtained here provide a foundation for rational antiviral design targeting critical N-RNA interactions against BoDV-1 and related viruses.

## RESULTS

### Protein purification and structural analysis of full-length BoDV-1 N complexes

Full-length BoDV-1 N with an N-terminal hexahistidine tag was expressed in *Escherichia coli* and purified using affinity chromatography followed by size exclusion chromatography (SEC) (fig. S1, A and B). Purified N protein eluted predominantly as a single peak during SEC, and the peak fraction exhibited a low absorbance ratio at 260 and 280 nm (*A*_260_/*A*_280_) (0.66), indicating minimal RNA inclusion. Cryo-EM analysis revealed that this fraction predominantly contained planar tetrameric complexes, which closely resembled the RNA-free N tetrameric structure previously reported using x-ray crystallography (fig. S1, C and D) (*6*).

In contrast, an earlier-eluting SEC fraction displayed a higher *A*_260_/*A*_280_ ratio of 1.36, suggesting the presence of substantial RNA. Cryo-EM analysis revealed structurally diverse N complexes (fig. S1, E to K). Single-particle cryo-EM identified complexes consisting of varying numbers of N subunits, including ring-like, S-shaped, and triangular configurations, ranging from tetramers to larger assemblies of up to 13 subunits (fig. S1K). Distinct densities attributable to RNA were observed in ring-like complexes containing more than five subunits. Other oligomeric forms, including some larger assemblies, did not exhibit any density corresponding to RNA-associated features seen in RNA-bound rings. Because individual oligomeric species such as the pentamer cannot be purified in isolation, we cannot directly validate their RNA content biochemically. Therefore, the term “RNA-free” indicates the absence of detectable RNA-related density in the corresponding cryo-EM maps, rather than definitive exclusion of trace or degraded RNA.

**Fig. 1.**
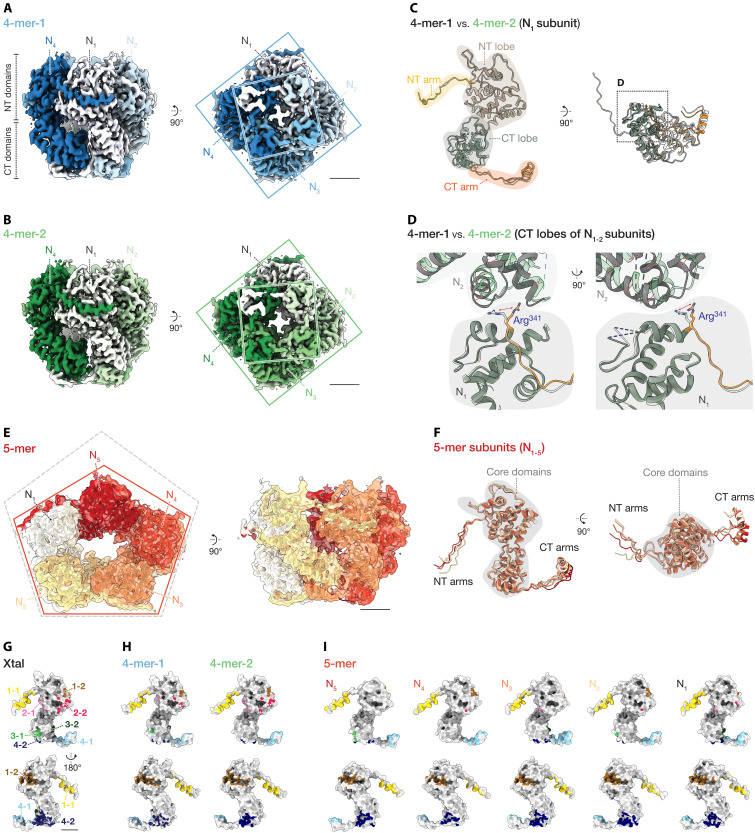
Structures of RNA-free, ring-like N tetramers and pentamers. (**A** and **B**) Cryo-EM reconstructions of tetrameric ring-like N complexes in two conformations: 4-mer-1 (A) and 4-mer-2 (B). Subunits are uniquely colored in each structure. Left panels: side views; Right panels: Bottom views. Square boxes indicate relative angles between the two lobes. (**C** and **D**) Structural comparison of N subunit models between 4-mer-1 (gray) and 4-mer-2 (domain-colored as in fig. S1A). Overall view of a subunit from inside the complex (left) and from the bottom (right), highlighting major differences in the C-terminal (CT) lobe and arm domains (C). Close-up of intersubunit region [dotted box in (C)] showing flipped side chain of Arg^341^ and relative displacement of CT lobes (red arrows) (D). (**E**) Cryo-EM reconstruction of 5-mer complex. Atomic models of N subunits (N_1-5_) in warm colors are superimposed on the cryo-EM map, forming an asymmetric pseudo-C5 structure (orange frame) compared to a regular pentagon (dotted gray frame). (**F**) Comparison of individual subunits in the pentamer, illustrating conserved core domains (gray) and divergent NT and CT arms. (**G** to **I**) Intersubunit interactions in crystal structure (Xtal) (G), tetramers (4-mer-1 and 4-mer-2) (H), and pentamer (5-mer) (I). Surface representation of asymmetric subunit models with interface clusters color-coded in four distinguishable pairs: (i) N-terminal arm (1-1: yellow) and a shallow groove on N-terminal lobe (1-2: brown); (ii) side area of N-terminal lobe (2-1: pink, 2-2: red); (iii) side area of C-terminal lobe (3-1: light green, 3-2: dark green); and (iv) C-terminal arm (4-1: light blue) and a pocket on C-terminal lobe (4-2: dark blue). These cluster numbers (1 to 4) correspond to the four types of N-N interaction interfaces described in the text. The comparison shows conserved interfaces in (1) and (4) and divergent interfaces in (2) and (3). Scale bars, 20 Å.

**Fig. 2.**
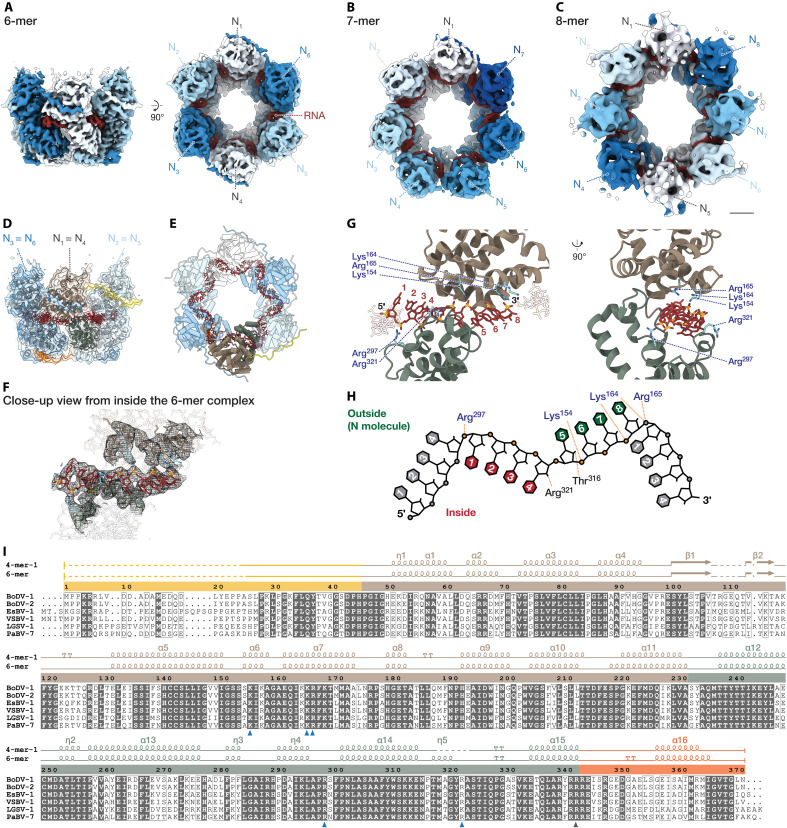
Structures of ring-like N-RNA complexes and N-RNA interactions. (**A** to **C**) Cryo-EM structure of the 6-mer (A), 7-mer (B), and 8-mer (C). Asymmetric N subunits are uniquely colored (white to blue), and RNA is shown in red. Scale bar, 20 Å. (**D**) Atomic model of 6-mer in ribbon representation. (**E**) Tube representation of 6-mer model, highlighting gear-like RNA structure positioned inside the ring (red). (**F**) Cryo-EM density of the 6-mer shown in mesh representation, centered on the RNA-binding cleft viewed from inside the 6-mer ring. The RNA density is highlighted together with the superposed atomic model displayed in stick representation. (**G**) Close-up of N-RNA interface, showing key basic residues involved in binding eight RNA nucleotides. Side chains are depicted as sticks and labeled. (**H**) Schematic of RNA configuration and N-RNA interactions. Inward-facing (red) and outward-facing (green) RNA bases and phosphate groups (orange) are illustrated. Putative hydrogen bonds and electrostatic interactions between N residues and RNA are shown as dashed lines. (**I**) Amino acid sequence alignment of the N from members in the genus *Orthobornavirus*: BoDV-1 (UniProt: UPO09291.1), Borna disease virus 2 (BoDV-2, UniProt: YP_009268917.1), estrildid finch bornavirus 1 (EsBV-1, UniProt: YP_009505423.1), variegated squirrel bornavirus 1 (VSBV-1, UniProt: YP_009269413.1), Loveridge’s garter snake virus 1 (LGSV-1, UniProt: YP_009055058.1), and parrot bornavirus 7 (PaBV-7, UniProt: YP_009268899.1). Conserved and similar residues are highlighted with filled and hollow boxes. Arrowheads indicate RNA binding basic residues (blue), and residues contributing to intersubunit distances (gray). Secondary structure assignments are shown for RNA-free 4-mer-1 (PDB ID: 9JZJ) and the RNA-bound 6-mer (chain B, PDB ID: 9JZL). Solid lines denote modeled regions, dotted lines indicate unmodeled regions, and dots mark alignment gaps. α helices and β sheets are indicated by coils and arrows, respectively. Domains in (D) to (F) and (I) are color-coded as in fig. S1A.

In total, we identified 16 distinct N complex types, of which 15 were reconstructed to three-dimensional (3D) structures at resolutions ranging from 2.8 to 8.6 Å, excluding a nonameric ring-like structure (figs. S1, E to K, and S2 and table S1). For reconstructions better than 4-Å resolution, atomic models were built on the basis of well-resolved protein backbones, with side-chain densities frequently visible, allowing for detailed interpretation of molecular architecture (Figs. 1 and 2 and figs. S2 and S3).

Furthermore, cryo-EM data showed considerable structural heterogeneity, with continuous conformational variability observed in each oligomeric species (movie S1). This heterogeneity suggests that N molecules and their assemblies have intrinsic dynamic flexibility.

Similar oligomeric assemblies were observed when N protein was expressed in human cells (fig. S1, L and M). The cryo-EM analysis of a human embryonic kidney–293T (HEK293T) cell–expressed sample revealed well-resolved tetrameric, pentameric, and hexameric particles plus a range of higher-order oligomers. Among these higher-order assemblies, we also identified elongated S-shaped complexes likely having 10 full-length N subunits and ring-like or loop-like particles containing nine or more subunits. These findings demonstrate that a broad range of N oligomeric forms can arise during host cell expression, illustrating the capacity of BoDV-1 N to achieve diverse assembly states beyond those captured in bacterial expression systems.

### Overall structures of RNA-free, ring-like N tetramers and pentamers

Crystal structures may differ from solution-state conformations due to crystal packing constraints. To determine whether cryo-EM structures of RNA-free N complexes matched the previously reported crystal structure, we examined their architecture and conformational variability in solution.

Cryo-EM analysis revealed two slightly different conformations of the tetrameric N complex, exhibiting cyclic 4-fold (C4) symmetry (Fig. 1, A and B). Both conformations maintained a conserved core architecture, characterized by interactions mediated by N- and C-terminal arm domains between adjacent subunits. The primary structural difference between these two conformations was observed in the relative orientation of the C-terminal domains, which differed by an ~8.5° rotational shift (fig. S3A). Structural alignment demonstrated high similarity between one cryo-EM tetramer conformation (class 1: “4-mer-1”) and the crystal structure at the monomeric level, with a backbone root mean square deviation (RMSD) of 0.70 Å. However, the crystal structure appeared slightly more compact, likely due to packing-induced constraints (fig. S3B). This similarity suggests that crystal packing effects alone do not shape the tetrameric arrangement observed in the crystal structure but reflect an inherently stable configuration adopted by the protein in solution.

The second tetrameric conformation (class 2: “4-mer-2”) exhibited an RMSD of 1.27 Å compared to the 4-mer-1 structure. In 4-mer-2, residue Arg^341^ at the boundary between adjacent C-terminal lobes adopted a different rotamer conformation, resulting in notable structural divergence in intersubunit distances (Fig. 1, C and D, and fig. S3C).

We also identified and reconstructed the 3D structure of an RNA-free ring-like pentameric N complex (“5-mer”) at an overall resolution of 3.8 Å. This structure displayed ring-like morphology with pseudo-C5 symmetry (Fig. 1E). Similar to the tetramers, pentameric subunits interact via their N- and C-terminal arms, with high structural conservation observed in the core domain (residues 44 to 341). Despite this conservation, the 5-mer exhibits intrinsic asymmetry, with subtle positional deviations among subunits (Fig. 1F).

In both tetrameric and pentameric configurations, several regions displayed reduced map density indicative of intrinsic flexibility or disorder (fig. S3, D to I). Specifically, residues at approximately positions 1 to 24 and 316 to 323 consistently show low local resolution, consistent with previous crystallographic observations. In addition, the loop in protruding β strands (residues ~106 to 111) could not be modeled due to insufficient map density.

Local resolution of the maps and *B*-factors of the models were also poor for residues 38 to 42, which form the connecting part of the N-terminal arm and N-terminal lobe, residues 120 to 127, a relatively long loop region largely exposed to the solution, and residues 347 to 354 surrounding the linker region between the C-terminal lobe and C-terminal arm helix. These findings suggest enhanced local mobility in solution.

The high *B*-factor of the N-terminal arm was particularly pronounced in one subunit (N_5_) of the pentameric structure, contributing to asymmetry and local heterogeneity (fig. S3F). This structural asymmetry and localized disorder likely reflect transient intersubunit interactions and inherent plasticity of these oligomeric complexes. Collectively, these observations highlight the dynamic behavior of N assemblies, which may facilitate transitions required for nucleocapsid assembly.

### N-N interactions in tetramers and pentamers

The previously reported crystal structure of the RNA-free tetrameric N complex identifies four distinct N-N interaction clusters crucial for maintaining complex stability. These interaction clusters comprise: (i) hydrophobic and electrostatic interactions between the N-terminal arm of one subunit (N*_n_*) and a shallow groove on the N-terminal lobe of the adjacent subunit (N_*n+*1_), (ii) polar interactions between the N-terminal lobes of adjacent subunits (N*_n_* and N_*n+*1_), (iii) polar interactions between the C-terminal lobes of adjacent subunits (N*_n_* and N_*n*−1_), and (iv) hydrophobic and electrostatic interactions linking the C-terminal arm helix of one subunit (N*_n_*) with the C-terminal lobe of the neighboring subunit (N_*n*−1_) (Fig. 1G).

To assess modes of N-N interactions in our RNA-free N complexes, we analyzed interfaces in tetrameric and pentameric assemblies (Fig. 1, G to I). The 4-mer-1 shows N-N interactions consistent with those observed in the crystal structure, with all four interaction clusters preserved. However, 4-mer-2 displays reduced contacts between adjacent C-terminal lobe interfaces, which results from a rotational shift and torsion in domain orientation (Fig. 1H).

In the pentameric complex, interactions mediated by N- and C-terminal arm domains were maintained, as in tetramers. However, polar contacts between lobe domains are generally reduced and vary notably across subunits, reflecting inherent asymmetry and structural plasticity (Fig. 1I).

These cryo-EM observations indicate that the stability of RNA-free N complexes is primarily governed by interactions involving terminal arm domains. More transient polar interactions between lobe domains occur across solvent-exposed, loosely packed interfaces, suggesting a dynamic equilibrium in solution. This behavior parallels observations in ring-like N-RNA complexes of HRSV (*10*) and interstrand N-N interactions in EBOV helical nucleocapsids (*16*).

### Structures of ring-like N-RNA complexes and N-RNA interactions

Previous structural studies on mononegavirus N-RNA complexes revealed distinct RNA binding modes: RNA is located externally in complexes in the families *Paramyxoviridae*, *Pneumoviridae*, and *Filoviridae* but encapsidated internally in *Rhabdoviridae* (fig. S4, A to D). Despite these differences in RNA positioning, a conserved feature among mononegaviruses is that RNA binds to a cleft formed between the N- and C-terminal lobes of the N molecule. However, for BoDV-1, precise positioning has remained controversial, with reports suggesting either an external location opposite the cleft (*6*) or an internal binding mode in the cleft (*28*).

To clarify the RNA binding mode of BoDV-1, we analyzed BoDV-1 hexameric, heptameric, and octameric N-RNA complexes, termed “6-mer,” “7-mer,” and “8-mer,” respectively. These structures demonstrate conclusively that the RNA strand resides in the ring-like complexes and binds to the interlobe cleft, a mode previously observed only in *Rhabdoviridae* (Fig. 2, A to E, and fig. S4, D and E). The BoDV-1 N-RNA complex features a gear-like round structure with abrupt changes in RNA backbone curvature, also similar to rhabdoviruses, but distinct from other mononegaviruses (Fig. 2E and fig. S4, A to E).

Notably, BoDV-1 N exhibits unique RNA binding stoichiometry, with each N subunit binding eight nucleotides arranged in a sharply bending “4-bases-inside, 4-bases-outside” configuration. This mode is unprecedented in the order *Mononegavirales* (Fig. 2, F to H).

Consistent with other mononegaviruses, RNA binding in BoDV-1 N is predominantly electrostatic, mediated by interactions between basic amino acid residues and RNA phosphate groups (Fig. 2, F to H). Sequence alignment in the genus *Orthobornavirus* shows that these residues—including Lys^154^, Lys^164^, Arg^165^, Arg^297^, and Arg^321^—are highly conserved (Fig. 2I). These conserved interactions indicate that N primarily recognizes RNA via its phosphate backbone and local conformation (Fig. 2G). This backbone-dependent RNA recognition provides a mechanistic explanation for the previously reported sequence preference in BoDV-1 N-RNA interactions. The apparent specificity likely arises from structural complementarity between the RNA backbone and the binding cleft of N, rather than from direct base-specific contacts (*28*).

### Local conformational transition of N upon RNA binding

Mononegavirus N molecules cooperatively transition from monomeric RNA-free states to oligomeric RNA-bound states during virus assembly (fig. S4, F to O). Therefore, it is crucial to examine how N molecules undergo conformational changes upon RNA binding to identify the mechanism of nucleocapsid assembly.

**Fig. 3.**
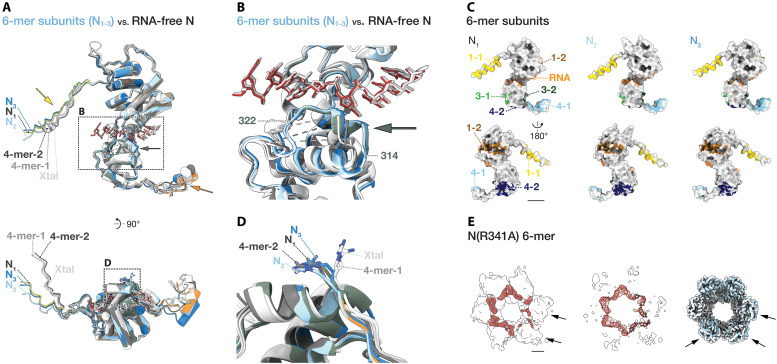
Structural features of RNA-bound N hexamer and effects of the R341A mutation. (**A**) Structural comparison of N subunits from RNA-free tetramers (crystal structure, 4-mer-1, 4-mer-2, gray colors) and those from 6-mer (colored) shown in tube representation. Arrows indicate major structural differences, color-coded by domain for the N_1_ subunit. Dashed boxes highlight the RNA binding cleft [close-up in (B)] and the intersubunit interface of the C-terminal lobes [close-up in (D)]. (**B**) Close-up view of the RNA-binding cleft in ribbon representation, illustrating a local structural transition in the C-terminal loop region to a helix (residues 314 to 322) upon RNA binding. (**C**) Intersubunit interfaces in 6-mer complex. Surface representation of asymmetric subunit models, with interface clusters color-coded as in Fig. 1G. The RNA interface highlighted in orange. (**D**) Close-up of Arg^341^ side chains of 6-mer complex and RNA-free tetramers, showing flipping-in conformation, similar to that observed in 4-mer-2 (Fig. 1D). (**E**) Left: The full cryo-EM map of the N(R341A) 6-mer. The density colored in red was obtained from a difference map generated by subtracting a 7.83-Å resolution map calculated from the fitted model of wild-type 6-mer (chains A and R, multiplied six times) using the *molmap* function in UCSF ChimeraX from the N(R341A) 6-mer map. This density is located near the RNA binding cleft. The difference density contains a particularly weak signal corresponding to RNA associated with only two adjacent subunits (arrows). Middle: The difference map, with the RNA model shown in stick representation. Right: Superposition of the core domains of a rigid-body–fitted N(R341A) 6-mer protein model with the wild-type 6-mer model shown in wire representation, showing substantial deviations in the spatial arrangement of two to three subunits (arrows). Scale bars, 20 Å.

To explore structural differences upon RNA encapsidation, we compared RNA-free and RNA-bound BoDV-1 N complex structures, 4-mer-2 and 6-mer. The overall structure and relative orientation of N- and C-terminal lobe domains (residues 44 to 341) remain consistent between RNA-free and RNA-bound states, similar to observations in rhabdoviruses (fig. S4, I and N), excluding a hinge-like conformational change observed in paramyxoviruses and pneumoviruses (Fig. 3A and fig. S4, F, G, J, K, L, and O). Regions of poor local resolution, indicative of high local flexibility, are also similar between the two states (fig. S3, G to K). However, a local structural transition was observed in the loop region (residues 314 to 322) of the C-terminal lobe. This region, disordered in RNA-free complexes, becomes ordered and adopts a short helix with a 3_10_ geometry upon RNA binding (Fig. 3B). This loop-to-helix transition resembles that observed in filoviruses (*16*, *20*), suggesting a conserved RNA binding mechanism throughout the *Mononegavirales* (fig. S4, H, J, M, and O). The high-resolution cryo-EM map allowed unambiguous modeling of this helix, confirming the loop-to-helix transition as a definitive structural feature induced by RNA binding. Although this region does not consistently adopt a defined secondary structure in other mononegaviruses, its recurrent alignment parallel to the RNA supports its designation as the “RNA binding loop,” a structurally and functionally conserved feature in this order (fig. S4, P to T).

In BoDV-1 N-RNA complexes, intersubunit interactions primarily involve extended N- and C-terminal arm domains (Fig. 2D), with variations in angles and distances between subunits, analogous to RNA-free tetrameric and pentameric complexes (Fig. 3C). Compared to RNA-free tetramers, RNA-bound hexamer displays a wider ring architecture due to the outward swing of the arm domains upon RNA binding (Fig. 3A, bottom). This outward movement creates larger intersubunit gaps and reduces the contact surface area between the core domains (Figs. 1, G to I, and 3C). This is accompanied by a flip-in of the conserved Arg^341^ side chain toward the intersubunit interface, as also observed in 4-mer-2 complex (Figs. 1, D and H, and 3, C and D).

To further assess the contribution of Arg^341^ to these intersubunit rearrangements, we analyzed the Arg^341^Ala mutant of N, denominated N(R341A). The SEC profile resembled that of the wild type and yielded both a major tetrameric fraction and a broader, earlier-eluting fraction (fig. S5A). Negative-stain transmission electron microscopy (TEM) of both fractions revealed a tendency toward small aggregates (fig. S5, B to D), suggesting reduced stability of the complexes. Cryo-EM analysis of the mutant sample identified tetramers (“4-mer”), asymmetric tetramers (“asymmetric 4-mer”), adjacent tetramers (“4-mer x2 antiparallel”), hexamers (“6-mer”), and heptamers (“7-mer”), reconstructed at resolutions of 3.6, 7.0, 6.2, 7.8, and 7.8 Å, respectively. 4-mer was structurally closer to the wild-type 4-mer-1, as indicated by a smaller backbone RMSD (1.02 Å compared with 1.78 Å for 4-mer-2) and accordingly adopted an overall configuration in which the C-terminal domains remained closely positioned (fig. S5G). Among all N(R341A) assemblies, C-terminal domains remained comparatively well-defined and retained a closed arrangement, whereas N-terminal domains consistently exhibited relatively blurred density and locally reduced resolution (fig. S5, F, H, and I). The asymmetric 4-mer displayed a closed C-terminal domain but an open central channel formed by the four N-terminal domains compared with the 4-mer (fig. S5J). In hexameric and heptameric assemblies, RNA density was detectable but markedly weaker in two subunits, suggesting that altered intersubunit stability and geometry may hinder uniform engagement of RNA (Fig. 3E and fig. S5K). Consistent with these observations, the proportion of hexameric particles in the dataset was relatively low (fig. S5E), and subunits that adopted configurations deviating most from the wild-type arrangement corresponded to the same two to three subunits that displayed reduced RNA density in the 6-mer and 7-mer reconstructions (Fig. 3E and fig. S5K).

Collectively, these findings indicate that the conformational transition of BoDV-1 N upon RNA encapsidation primarily involves local restructuring of the RNA binding loop, while the overall oligomeric architecture remains largely preserved. The more open ring-like architecture observed in RNA-bound assemblies, characterized by increased intersubunit spacing and a broadened internal channel, appears to reflect intrinsic conformational flexibility of N that enables RNA accommodation.

### Conformational plasticity and RNA binding heterogeneity in hexameric N complexes

**Fig. 4.**
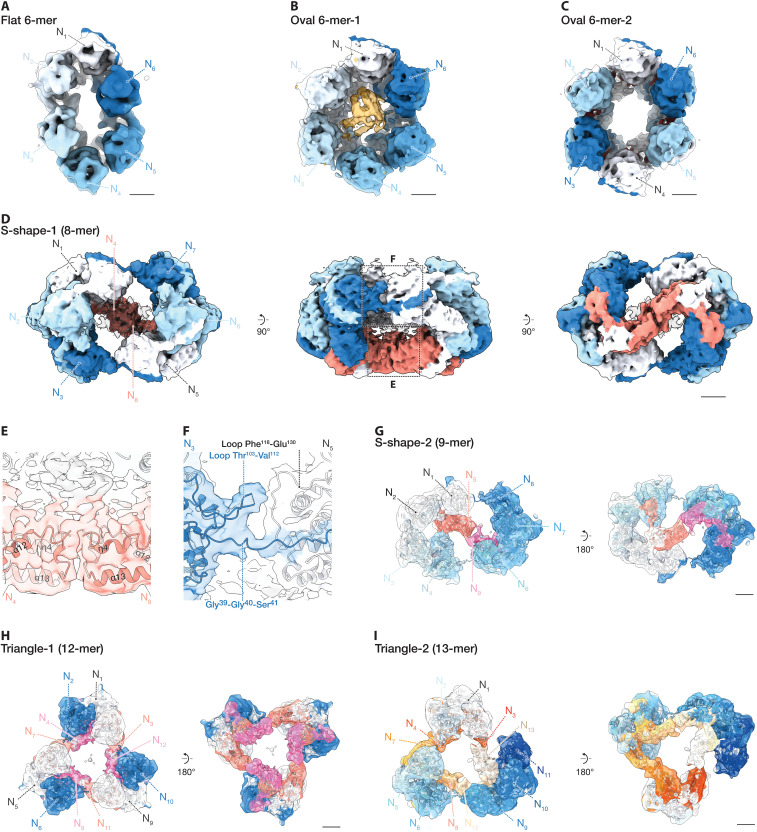
Diverse higher-order assemblies of BoDV-1 N: oval hexamers, S-shaped, and triangular complexes. (**A** to **C**) Cryo-EM reconstructions of oval-shaped hexameric N complexes: the most elongated ring-like complex (Flat 6-mer) (A), an oval complex with extra central density (Oval 6-mer-1) (B), and an oval complex with partial RNA density (Oval 6-mer-2) (C) reconstructed with C1, C1, and C2 symmetry, respectively. Asymmetric subunits are uniquely colored (white to blue) in all panels. (**D** to **I**) Cryo-EM reconstructions of N complexes composed of full-length N molecules bridged by N_CTD_ (C-terminal domain only). Each asymmetric subunit is color coded: full-length in blue tones and N_CTD_ in warm colors (e.g., orange or red). (D) “S-shape-1”: two trimeric units bridged by an N_CTD_ pair. (E) Close-up of the interface between an N_CTD_ pair, showing direct interaction at the bridging site. (F) Close-up of the interface formed by an N-terminal arm, highlighting a distinct intersubunit contact. (G) “S-shape-2”: a trimer and a tetramer bridged by an N_CTD_ pair, arranged asymmetrically. (H) “Triangle-1”: three dimers arranged in a triangle and bridged by three N_CTD_ pairs along each edge of the triangle. (I) “Triangle-2”: two dimers and a trimer bridged by three N_CTD_ pairs. Scale bars, 20 Å.

In addition to hexameric complexes displaying distinct RNA densities, we identified structurally variable hexameric ring-like assemblies that exhibit variable degrees of circularity (Fig. 4, A to C). The most elongated form, hereafter referred to as the “Flat 6-mer,” shows no detectable RNA density in the RNA-binding cleft, indicating that it represents an RNA-free state (Fig. 4A and fig. S6A). A second, more oval-shaped assembly (“Oval 6-mer-1”) contains additional density at its center (Fig. 4B). After classification and isolation of the relevant 3D class, densities corresponding to the N subunits were masked, allowing detailed examination of the central density. The isolated density exhibited clear α-helical characteristics, including a helical pitch consistent with that of a canonical α helix, indicating that it corresponds to a polypeptide. Rigid-body fitting of any domain of the N molecule did not yield a plausible placement, suggesting that the density does not correspond to these domains in their known folds. The ModelAngelo algorithm (*29*) predicted an α-helical structure for the well-resolved portion of the density, supporting the conclusion that it corresponds to a polypeptide (fig. S6, B and C). However, sequence assignment remained inconclusive, likely due to limited local detail in the map. A more interpretable cryo-EM map or higher-sensitivity mass spectrometry will be needed to determine the molecular origin of this protein component. This complex also lacks visible RNA in the canonical RNA binding cleft (Fig. 4B and fig. S6D). A third assembly, “Oval 6-mer-2,” exhibits weak but discernible RNA density in the cleft, suggesting the possibility of partial RNA occupancy or transient interactions (Fig. 4C and fig. S6E). These structural variations collectively demonstrate that hexameric N complexes can adopt similar global architectures regardless of their RNA-bound state.

Cryo-EM density corresponding to the RNA binding loop varied among the three hexamers. In the RNA-free Flat 6-mer, this region appears poorly resolved, consistent with increased conformational flexibility or disorder, as also observed in RNA-free tetramers and pentamers (fig. S6A). In contrast, the Oval 6-mer-1 shows density indicative of a helical secondary structure, although some adjacent loop regions remain less well defined (fig. S6D). The RNA-bound Oval 6-mer-2 exhibits a well-ordered helical conformation similar to that in both Oval 6-mer-1 and the fully RNA-bound 6-mer complex (fig. S6, E and F).

These findings suggest that the helical conformation in the RNA binding loop region is not preformed but is instead induced upon interaction with either an RNA molecule, as in Oval 6-mer-2, or with another protein component, as observed in Oval 6-mer-1.

### Discovery of noncanonical oligomeric states of N involving truncated C-terminal domains

Mononegavirus N molecules typically assemble into ring-like or helical structures. While these canonical assemblies are well-characterized, alternative oligomeric states remain poorly understood. To explore the full range of assembly possibilities, we sought to identify and characterize highly unconventional oligomeric assemblies of BoDV-1 N that deviate markedly from known architectures.

In addition to canonical ring-like structures, our cryo-EM analysis revealed highly unconventional RNA-free complexes exhibiting S-shaped and triangular morphologies, designated “S-shape-1,” “S-shape-2,” “Triangle-1,” and “Triangle-2” (Fig. 4, D to I). These complexes contain truncated N subunits composed solely of the C-terminal domain (N_CTD_). These act as bridging subunits connecting clusters of full-length N molecules, a configuration not previously reported for other mononegaviruses.

Structural analysis identifies two intersubunit interfaces in these assemblies (i) a hydrophobic surface patch that is typically buried in full-length N becomes exposed and mediates interaction between N_CTD_ subunits (Fig. 4E and fig. S7D) and (ii) polar loop regions (residues 103 to 112 and 118 to 130) that form an additional contact site, potentially involving electrostatic or hydrogen bonding interactions (Fig. 4F). These interfaces are maintained in all distinct, novel RNA-free assemblies.

The cryo-EM density map of the octameric S-shape-1 complex shows that all full-length subunits are connected via N-terminal arm domains (Fig. 4F). Notably, the connection between the most spatially distant subunits features a highly flexible Gly-Gly-Ser sequence (residues 39 to 41), located between the N-terminal arm and the core lobe of N, which permits a pronounced bend between domains (Fig. 4F and fig. S7, E and F). The resulting modular architecture, with distinct clusters bridged by full-length subunits, closely resembles that observed in tetrameric and pentameric assemblies. This spatial correspondence suggests a domain-swapping mechanism (fig. S7G), as observed in other mononegaviruses, including vesicular stomatitis Indiana virus (*7*), MV (*11*), human parainfluenza virus 5 (*30*), and mumps virus (MuV) (*31*).

The SDS–polyacrylamide gel electrophoresis (SDS-PAGE) analysis of highly concentrated, purified N samples from *E. coli* revealed a prominent ~15-kDa band corresponding to the N_CTD_ fragment (fig. S7A). Liquid chromatography tandem mass spectrometry (LC-MS/MS) confirmed that this fragment originates from cleavage at the boundary region between the N- and C-terminal lobes (fig. S7B). To evaluate the stability of each domain in isolation and to determine whether the electrophoretic mobility of this species corresponds to that of individual domains, we expressed and purified the isolated N-terminal domain (N_NTD_, residues 1 to 230) and N_CTD_ (residues 231 to 370). The N_NTD_ showed very poor expression and was prone to degradation, consistent with the absence of a stable N-terminal fragment in full-length N preparations. In contrast, the N_CTD_ was robustly expressed, and the SDS-PAGE of the Ni–nitrilotriacetic acid elution fraction revealed a ~15-kDa band whose mobility resembled that of the species observed during purification of full-length N. These observations indicate that the ~15-kDa band corresponds to a C-terminal domain-derived fragment (fig. S7C).

In addition, 3D reconstructions revealed two distinct assemblies composed of side-by-side tetramers (“4-mer x2 parallel” and “4-mer x2 antiparallel”) (fig. S7, H and I). In addition to these assemblies, 2D class averages also revealed cases in which a pentamer and a tetramer, or a hexamer and a tetramer, appear nearby when expressed in both *E. coli* and human cells (fig. S1, I and M). These multimeric arrangements further support the idea that flexible interdomain linkers enable architectural transitions and may facilitate domain-swapping in BoDV-1 N.

### Virological importance of RNA binding basic residues

IB formation is a hallmark of many mononegavirus infections. In BoDV-1–infected cells, these structures localize to the nucleus. The coexpression of N and P is sufficient to induce formation of IB-like structures in most mononegaviruses (*32*–*36*), including BoDV-1 (*37*). While our cryo-EM structures reveal how N recognizes RNA and assembles into ring-like oligomers, they do not clarify whether RNA binding by N is strictly required for subsequent steps in the replication cycle, such as RNA synthesis and, more specifically, formation of IBs.

To address this issue, we performed structure-guided alanine-scanning mutagenesis targeting four key RNA-contacting residues of N: Lys^154^, Lys^164^, Arg^297^, and Arg^321^. Resulting mutants—N(K154A), N(K164A), N(R297A), and N(R321A)—were evaluated using a BoDV-1 minireplicon system and a nuclear IB reconstitution assay.

**Fig. 5.**
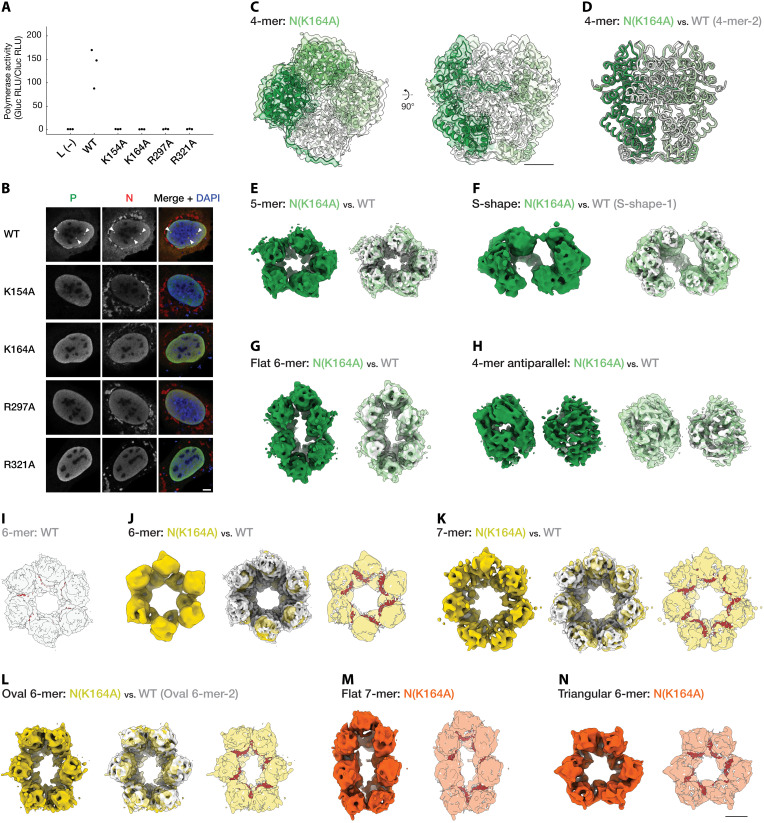
Structure-guided mutational assays. (**A**) Polymerase activity assessed by minireplicon assay using wild-type (WT) and mutant N (*n* = 3). All mutations caused a marked reduction in activity. (**B**) IB reconstitution assay (*n* = 3) showing subcellular localization of WT or mutant N co-expressed with WT P. Left: P (gray). Center: N (gray). Right: Merged images (N: red, P: green, and nuclei: blue). White arrowheads highlight nuclear IB-like structures co-occupied by N and P. Scale bar, 5 μm. (**C** to **N**) Cryo-EM reconstructions of N(K164A) reveal diverse oligomeric states, with colored maps indicating WT-like (green), WT-like lacking RNA density (yellow), and WT-distinct (orange) structural classes. (C) 4-mer structure with an atomic model overlay. Subunits are uniquely colored. (D) Superposition of N(K164A) and WT 4-mer models in wire representation, showing high overall similarity. Additional assemblies: 5-mer (E), S-shape (F), Flat 6-mer (G), and 4-mer x2 antiparallel (H). Left: Mutant maps. Right: Map-to-map comparisons with WT (white). (I) WT 6-mer map with rigid-body–fitted model (N: chain B, white; RNA: chain R, red). The fitted model confirms the presence of RNA in the binding cleft, serving as a reference for RNA localization in subsequent comparisons. Ring-like assemblies: 6-mer (J), 7-mer (K), and Oval 6-mer (L). Left: Mutant maps. Center: Map-to-map comparison with WT (white). Right: Map-to-model comparison using WT N-RNA complex model (protein: white, RNA: red). Core domains of WT 6-mer chain B were rigid-body–fitted to assess RNA occupancy. In all structures, the RNA binding cleft lacked corresponding density, and RNA models protrude from the map, indicating loss of RNA. N(K164A)-specific Flat 7-mer (M) and Triangular 6-mer (N). Left: Mutant maps. Right: Map-to-model comparison using WT model (protein: white, RNA: red). The RNA binding cleft also lacked density, as evidenced by RNA models protruding outside the cryo-EM map. Scale bars, 20 Å.

The minireplicon assay revealed that all four mutants were completely defective in supporting viral RNA synthesis, indicating that these basic residues are essential for the replication function of N (Fig. 5A). Furthermore, each mutation abolished formation of nuclear IB-like structures upon coexpression with the viral P in human cells (Fig. 5B).

These findings indicate that RNA binding by N is required not only for its replication function but also for forming biomolecular condensates involved in nucleocapsid assembly.

### Stable ring-like assemblies in the absence of RNA

Nucleocapsid assembly has conventionally been considered RNA dependent. However, the specific contribution of RNA to assembly of ring-like N oligomers in mononegaviruses, including BoDV-1, remains unclear. To investigate whether BoDV-1 N can assemble into ring-like structures in the absence of RNA, we analyzed mutants with diminished RNA binding capacity using single-particle cryo-EM.

We expressed and purified BoDV-1 N mutants, N(K154A), N(K164A), and N(R321A) in *E. coli* (fig. S8A). The RNA content of purified mutant complexes was estimated using their *A*_260_/*A*_280_ ratios, measured from SEC fractions corresponding to the elution range of RNA-bound wild-type complexes. These ratios were 1.22 for N(K154A), 0.67 for N(K164A), and 1.57 for N(R321A) compared to 1.36 for wild-type N. Although values vary among mutants, they suggest different levels of RNA association.

Subsequent cryo-EM analysis focused on the N(K164A) mutant, presumed to have the lowest RNA binding affinity. 3D reconstructions revealed various oligomeric assemblies that closely resemble wild-type structures but lack detectable RNA (Fig. 5, C to N, and fig. S8, B to E). The RNA-free tetrameric structure closely matched the wild-type 4-mer-2 complex, with an RMSD of 0.62 Å, confirming that the Lys^164^Ala mutation does not disrupt protein folding (Fig. 5, C and D).

Notably, RNA-free heptameric ring-like complexes, absent in wild-type preparations, appear with other ring-like complexes exhibiting broader variations in circularity (Fig. 5, M and N). Triangular N_CTD_-bridged complexes were not detected, possibly due to their low abundance.

These cryo-EM data demonstrate that BoDV-1 N has an inherent capacity to form structurally diverse, RNA-free oligomers, including expanded ring-like architectures. This implies that these rings arise from the intrinsic self-assembly capacity of the N molecule, rather than from RNA-mediated nucleation followed by dissociation, reinforcing the conclusion that RNA binding is not a prerequisite for ring-like complex formation.

## DISCUSSION

**Fig. 6.**
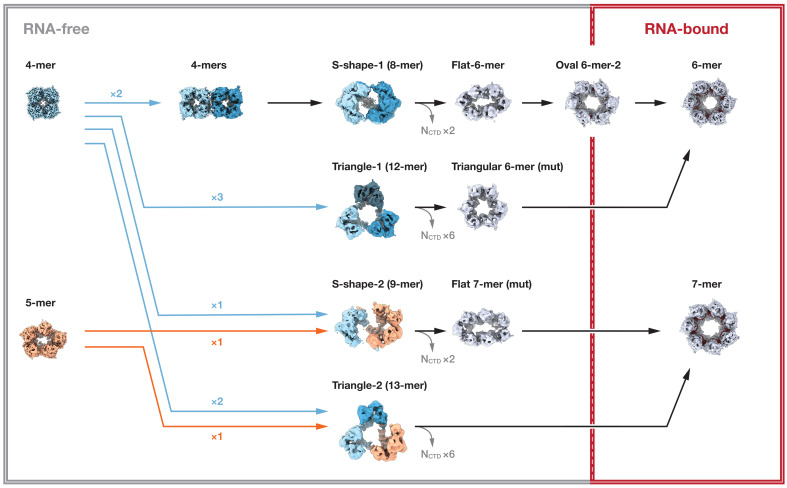
A model of ring-like N-RNA complex assembly. Cryo-EM analysis of BoDV-1 N protein reveals diverse oligomeric states, representing potential intermediate stages in nucleocapsid assembly. The model begins with RNA-free tetrameric or pentameric complexes, which serve as initial oligomerization cores. These structures may undergo fusion and reorganization, giving rise to asymmetric intermediates such as S-shaped and triangular assemblies, involving domain swapping and transient N_CTD_ bridging. Subsequent removal of N_CTD_ and structural realignment of arm domains lead to formation of ring-like hexamers and heptamers. Upon RNA binding, a local loop-to-helix transition is induced in the C-terminal lobe (residues 314 to 322), stabilizing RNA encapsidation. The final structure is a fully assembled RNA-bound hexamer, with coordinated intersubunit arm interactions and well-positioned RNA in the interlobe clefts. This model illustrates the stepwise and flexible nature of BoDV-1 nucleocapsid assembly, integrating both RNA-free self-assembly and RNA-guided structural stabilization.

Before this study, the BoDV-1 N-RNA complex was the only uncharacterized structure among human mononegavirus families, highlighting the significance of these findings. Our single-particle cryo-EM and functional analyses of the BoDV-1 N-RNA complex have addressed this gap in the structural catalog of N molecules in the order *Mononegavirales*. Our findings highlight evolutionary conservation across the *Mononegavirales* while also revealing lineage-specific adaptations that advance our understanding of nucleoprotein biology. Extensive image classifications enable visualization of various conformational states in solution, capturing both RNA-free and RNA-bound forms, including tetrameric, pentameric, hexameric, larger ring-like assemblies, and noncanonical arrangements. These states may fail to be detected by x-ray crystallography, which favors homogeneous, well-ordered populations due to crystal packing. In contrast, cryo-EM reveals a broader conformation ensemble that more closely reflects physiological diversity. The resolved structures define key architectural principles of N oligomerization and correlate directly with RNA encapsidation efficiency, as supported by structure-guided mutational analyses. Collectively, they illustrate a previously unrecognized, stepwise assembly pathway for N-RNA complexes (Fig. 6), in which N first oligomerizes independently of RNA, forming intermediates that subsequently recruit RNA. Notably, this contrasts with canonical models in which RNA initiates and guides N assembly and instead supports a regulated pathway in which RNA-free intermediates act as checkpoints for genome encapsidation.

### Ring-like N assembly and its implications

Ring-like N-RNA complexes have been observed in several mononegaviruses, including rhabdoviruses (*7*, *8*) and pneumoviruses (*10*), before their biological relevance was fully understood. Subsequent studies confirmed that these assemblies share the same RNA binding mode as helical nucleocapsids, demonstrating that they are not artifacts but instead represent the fundamental principle of viral nucleocapsid formation (*38*–*41*). Cryo-EM analyses of MuV N-RNA complexes revealed that N adopts nearly identical structures in ring-like and helical complexes (RMSD of ~0.83 Å) and that ring-like structures can transition into helices upon exposure to cell-derived impurities (*31*). This finding supports the idea that ring-like assemblies are structurally representative and potentially dynamically interconvertible intermediates. The cryo–electron tomography of HRSV-infected cells and virions further confirmed the presence of these ring-like assemblies in a native setting (*42*), highlighting their potential relevance during viral replication.

Our cryo-EM structural data demonstrate that BoDV-1 N forms stable ring-like assemblies, exhibiting key architectural features conserved among mononegavirus N proteins. These include extended N- and C-terminal arms that mediate intersubunit interactions. The basic ring-like architecture is preserved across a spectrum of oligomeric forms, including hexamers, heptamers, and octamers, reflecting structural plasticity rather than a single dominant configuration (Fig. 2, A to C). The observed variability in ring size and symmetry, together with the flexibility of polar interfaces and domain-swapping termini (Fig. 3, A to C), suggests that these assemblies are modular and reversible intermediates capable of transitioning from ring-like to filamentous forms. This plasticity may support dynamic reorganization of the nucleocapsid during different stages of the viral life cycle—such as replication, transcription, and packaging—and may reflect a conserved strategy across mononegavirus families.

The current structures represent fundamental “mini-nucleocapsid cores” rather than complete viral nucleocapsids. Determining the structure of larger supercomplexes capable of incorporating full-length genomic RNA remains a future challenge. Bornaviruses replicate chronically in the host cell nucleus in a unique manner, producing extremely low viral titers (*43*). The amount of infectious virus released typically falls below detection limits, in stark contrast to other mononegaviruses that cause acute infections and release between 10,000 and millions of infectious particles per milliliter. Consequently, further technological developments—including improved viral purification, concentration methods, and nucleocapsid reconstitution techniques—are required to define the complete native nucleocapsid structure and its biological roles.

### Working assembly model of ring-like N-RNA complexes

Our cryo-EM analyses revealed unexpected structural diversity in BoDV-1 N assemblies, including symmetric and asymmetric complexes with varying RNA occupancy. These findings highlight a remarkable degree of conformational plasticity and suggest that the assemblies captured here may correspond to structurally relevant configurations with potential implications for nucleocapsid formation. On the basis of the oligomeric states observed, ranging from RNA-free tetramers to RNA-bound hexamers and heptamers, we outline a working assembly model for BoDV-1 N-RNA complexes (Fig. 6 and movie S2).

In this model, BoDV-1 N initially forms RNA-free tetramers or pentamers. These assemblies act as precursors for subsequent fusion events, forming RNA-free S-shaped or triangular complexes involving the N_CTD_ and domain swapping of N-terminal arm domains. The subsequent removal of the N_CTD_, along with domain swapping of the C-terminal arm domain, may facilitate formation of RNA-free hexameric and heptameric ring-like complexes.

Upon interaction with RNA, potentially facilitated by a chaperone-like polypeptide occupying the central channel, a local structural rearrangement occurs in the RNA binding loop (residues 314 to 322), transitioning from a disordered state into a structured helix (Fig. 3B and fig. S6, A and D to F). This transition stabilizes RNA encapsidation in the interlobe cleft.

Intersubunit interactions mediated by N- and C-terminal arm domains remain largely conserved between RNA-free and RNA-bound forms. However, RNA binding is associated with outward displacement of arm domains and reduced numbers of core domain contacts, resulting in more open and flexible ring-like complexes, suitable to accommodate RNA strands. Electrostatic contacts formed by several charged residues, including Arg^341^, contribute to tuning the spacing between adjacent lobes (Fig. 3, A to D). The structural analysis of Arg^341^Ala mutant provides additional insight into the structural role of Arg^341^. The increased prevalence of asymmetric tetramers and the partial loss of RNA density in the ring-like hexameric and heptameric assemblies suggest that Arg^341^ contributes to maintaining appropriate interdomain spacing required for stable assembly.

Our structure-guided mutational analyses further support the notion that RNA binding occurs after formation of ring-like N oligomers. Mutations that disrupted RNA interaction do not prevent assembly of N complexes observed in the wild type (Fig. 5, C to L), indicating that oligomerization can proceed independently of RNA binding. This implies a sequential assembly pathway in which N oligomerization precedes RNA encapsidation, allowing for regulated nucleocapsid formation. Such a mechanism could prevent premature or nonspecific RNA binding, ensuring that only properly assembled N oligomers participate in genome encapsidation.

Together, the RNA-free and RNA-bound assemblies described here broaden the structural landscape of BoDV-1 N. Although these forms suggest possible routes of oligomer transformation, further work will be required to determine how they relate to nucleocapsid formation. The N_CTD_-mediated bridging observed in some RNA-free complexes appears stoichiometrically inefficient, raising the possibility that a subset of N molecules is used during these rearrangements and that this process may help limit uncontrolled oligomerization or RNA engagement. Given that low–molecular weight N-related products have also been observed in other mononegaviruses (*15*, *44*), it would be useful to investigate whether truncated N contributes to N oligomerization in other members of the order. Several assemblies also remain difficult to interpret. For example, the biological significance, if any, of the additional central density in the Oval 6-mer-1 complex remains unclear and will require further characterization of its origin and structure. Moreover, because these forms have not yet been observed in infected cells, establishing their presence and relevance in a physiological context will be essential. Integrative approaches—including high-speed atomic force microscopy, biochemical probing, reconstitution assays, and infection-based imaging—will be key to clarifying how the assemblies characterized here fit into the dynamic pathway of nucleocapsid assembly.

### Comparative understanding of N-RNA complex structures and assemblies in negative-strand RNA viruses

Mononegavirus N shares a conserved core architecture and function, yet the BoDV-1 N-RNA complex exhibits notable distinctions compared to those of *Paramyxoviridae*, *Pneumoviridae*, *Filoviridae*, and *Rhabdoviridae*. Structurally, all N proteins comprise two lobed domains that encapsidate the RNA genome in a positively charged cavity, protecting the genome and serving as the template for viral polymerase activity. BoDV-1 N follows this general blueprint, with a conserved RNA binding loop that runs parallel to the RNA binding cleft and that is critical for securing the genome. In our structures, this loop becomes ordered and forms a short helix when bound to RNA or to a protein component but remains disordered in assemblies lacking these densities, providing a structural feature that correlates with ligand engagement (fig. S6). In BoDV-1 and filoviruses (*16*, *20*), this loop undergoes a conformational change upon RNA binding (Fig. 3, A and B, and fig. S4, H, J, M, and O), acting like a latch to lock the RNA in place. Mutations in the corresponding loop can impair RNA synthesis. In addition, the formation of an analogous α-helical element upon RNA engagement has been identified for the influenza virus nucleoprotein (family *Orthomyxoviridae*), outside the order *Mononegavirales*, thereby extending the reach of this conserved RNA binding architecture beyond this order (*45*–*47*). Furthermore, structural analyses reveal that RNA-bound influenza virus nucleoproteins can also adopt loop conformations in crystal structures (*48*) or recombinant nucleocapsid assemblies (*49*), indicating that this RNA binding motif exhibits notable conformational diversity and plasticity among negative-strand RNA viruses.

Although RNA recognition by N is nucleotide sequence independent in BoDV-1 and other members of the *Mononegavirales*, relying on a network of backbone contacts rather than recognizing specific base identities, RNA positioning and stoichiometry of N molecules vary among families (table S4). BoDV-1 N encapsidates eight nucleotides per subunit, in contrast to six in the *Paramyxoviridae* and *Filoviridae*, seven in the *Pneumoviridae*, and nine in the *Rhabdoviridae*.

Another point of divergence is how N transitions from an RNA-free state to an RNA-bound oligomer and the role of the P molecule in that process. In most mononegaviruses, newly synthesized N proteins readily bind nonspecific RNA and self-assemble, requiring chaperoning by P to prevent premature RNA encapsidation (*50*, *51*) and inappropriate oligomerization (*13*, *52*). BoDV-1 N, however, demonstrates exceptional stability in its RNA-free state and coexists with its RNA-bound state, as evidenced by our cryo-EM observations, suggesting reduced dependence on P for N oligomerization.

Evolutionarily, the mix of conserved and divergent features in N-RNA complexes among the *Mononegavirales* reflects their shared ancestry and long-standing functional constraints. The core structural framework appears to be ancient and highly conserved, highlighting the enduring requirement of genome encapsidation. At the same time, divergence in nucleotide stoichiometry and assembly patterns seems to have emerged gradually, likely driven by lineage-specific constraints and neutral drift, possibly emerging from transient stochastic variation in early assembly mechanisms.

The BoDV-1 N-RNA complex—when compared with its paramyxovirus, pneumovirus, filovirus, and rhabdovirus counterparts—provides a compelling case study of how different lineages in the *Mononegavirales* may have independently explored alternative structural solutions while preserving core functionality.

In conclusion, our comprehensive structural and functional analyses of the BoDV-1 N molecule not only address a critical knowledge gap regarding mononegavirus N structures but also provide insights into nucleocapsid assembly mechanisms. Our high-resolution cryo-EM structures of the BoDV-1 N-RNA complex reveal intricate details of binding interfaces and key residues mediating N-RNA interactions, identifying potential targets for antiviral therapeutics, particularly in the context of Borna disease. Furthermore, these findings contribute to a broader understanding of conserved N-RNA encapsidation and N-N interaction mechanisms throughout the order *Mononegavirales*. Our results provide not only a complete structural framework for mononegavirus N but also a model of modular, regulated encapsidation that may extend to other members of the order. By illuminating these fundamental viral processes, our work supports future targeted interventions against BoDV-1 and enhances our preparedness for emerging mononegaviruses by revealing conserved structural features that could be exploited for broad-spectrum antiviral strategies.

In addition, the current structural catalog is almost entirely limited to mammalian viruses, highlighting the need for further studies of viruses that infect nonmammalian animals as well as plants and fungi. Broader structural comparisons will advance our understanding of viral diversity and evolution and, hopefully, inform the development of targeted antiviral strategies. Such research would also support pandemic preparedness and One Health Initiative by identifying conserved and exploitable features across diverse viral taxa.

## MATERIALS AND METHODS

### Protein expression and specimen purification

The expression and purification of the full-length N protein (residues 1 to 370) of Borna disease virus 1 (the species *Mammalian 1 orthobornavirus*, UniProt: P0C796), produced in both *E. coli* and mammalian expression systems, and of the N-terminal domain (residues 1 to 230) and C-terminal domain (residues 231 to 370), produced exclusively in *E. coli*, were performed as described below.

For the *E. coli* expression system, an N-terminal hexahistidine-tagged N was expressed in Rosetta 2(DE3) pLysS competent cells (71403, Novagen Inc., Madison, WI, USA) through induction with 0.5 mM isopropyl β-d-thiogalactopyranoside at 16°C overnight. Pelleted cells were resuspended in a sonication buffer: Tris buffer [20 mM tris-HCl (pH 7.4) and 150 mM NaCl] containing 10 mM imidazole and a protease inhibitor cocktail (Nacalai Tesque Inc., Japan or Roche, Switzerland). After sonication, lysates were clarified by centrifugation at 20,000*g* for 10 min at 4°C. Then, supernatants were loaded onto a column containing TALON Metal Affinity Resin (Takara Bio Inc., Shiga, Japan). Resin was washed with sonication buffer, and histidine-tagged proteins were eluted with sonication buffer containing 300 mM imidazole. Samples were then applied to SEC using a Superdex 200 Increase10/300 GL column (Cytiva, USA) in tris buffer. Fractions were concentrated with Amicon Ultra Centrifugal Filters (Merck KGaA, Darmstadt, Germany) and stored at 4°C. Samples were subjected to SDS-PAGE, mass spectrometry, negative-stain TEM, and cryo-EM analysis.

For the mammalian expression system, HEK293T cells were transfected with a pCAGGS plasmid coding the full-length N protein with an N-terminal hexahistidine tag. Three days posttransfection, cell pellets were resuspended and gently mixed on ice for 15 min in lysis buffer: tris buffer containing 0.05% NP-40 substitute (Wako, 141-08321), a protease inhibitor cocktail (EDTA-free) (Roche, 11836170001), and 2 mM ribonucleoside-vanadyl complex (NEB, S1402S). Insoluble components were pelleted, and supernatant was collected and mixed with pre-equilibrated TALON Metal Affinity Resin (TaKaRa, 635502) and incubated on ice for 20 min, followed by two washes with binding buffer, tris buffer containing 10 mM imidazole, and pelleting of the resin. The resin was loaded onto a column, and histidine-tagged proteins were eluted with elution buffer: tris buffer containing 500 mM imidazole. Eluted samples were subjected to negative-stain TEM and cryo-EM to compare the assembly of N complexes in different expression systems.

### Negative-stain TEM

A 5-μl aliquot of a specimen was applied to freshly glow-discharged, carbon-coated 600-mesh copper grids (Gilder Grids Ltd., UK). After allowing samples to adhere for 1 min, excess liquid was gently blotted away using filter paper. Then, grids were immediately stained with three drops of 2% (w/v) phosphotungstic acid, adjusted to pH 7.0. After carefully removing excess staining solution, grids were air-dried at room temperature before imaging. Imaging was conducted on an HT-7700 transmission electron microscope (Hitachi High-Tech, Japan) at an acceleration voltage of 80 kV using an XR81-B charge-coupled device camera (Advanced Microscopy Techniques, USA).

### Cryo-EM specimen preparation and data acquisition

A 2- to 3-μl aliquot of specimen solution was applied twice onto glow-discharged Quantifoil R1.2/1.3 Gold 300 mesh grids [for wild-type N and N(K164A) from *E. coli*] or R1.2/1.3 Copper 300 mesh grids [for wild-type N from HEK293T cells and N(R341A) from *E. coli*] (Quantifoil Micro Tools GmbH, Germany). After blotting excess solution on the grids with filter paper, specimens were rapidly frozen in liquid ethane on a Vitrobot Mark IV (Thermo Fisher Scientific, USA). Cryo-grids were initially screened using a Glacios cryo-TEM (Thermo Fisher Scientific, USA) operated at an acceleration voltage of 200 kV, equipped with a Falcon4 direct electron detector at the Institute for Life and Medical Sciences, Kyoto University.

Cryo-EM data from *E. coli*–expressed wild-type N were collected on a prescreened cryo-grid on a Titan Krios cryo-TEM operated at 300 kV (Thermo Fisher Scientific, USA), equipped with a Cs corrector (CEOS GmbH, Germany) at the Institute for Protein Research, Osaka University. Image acquisition was performed using a beam-image shift scheme with a 3 × 3 hole configuration with two target positions per hole using the SerialEM software (*53*), with a nominal defocus range of −0.6 to −1.6 μm in energy-filtered transmission electron microscopy (EFTEM) nanoprobe mode. Images were acquired as 60-frame movies using a Gatan BioQuantum energy filter with a slit width of 20 eV and a K3 direct electron detector (Gatan Inc., USA) in the electron-counting and correlated double sampling (CDS) imaging mode. Movies (29,340) were acquired at a dose rate of 9.0 e^−^/pixel per s, a pixel size of 0.88 Å, and a cumulative exposure of 40 e^−^/Å^2^. Detailed imaging conditions are described in table S1.

Data from HEK293T cell–expressed N complexes were collected on a prescreened cryo-grid using the same Glacios cryo-TEM system. Image collection was conducted using aberration-free image shift capability managed in EPU software (Thermo Fisher Scientific, USA), with a nominal defocus range of −0.8 to −1.8 μm in nanoprobe mode with TIFF compression enabled. In total, 7344 compressed TIFF movies were acquired at a dose rate of 6.1 e^−^/pixel/s, a pixel size of 0.925 Å, and a cumulative exposure of 50 e^−^/Å^2^.

Data for *E. coli*–expressed N(R341A) were collected using the same microscope platform as HEK293T cell–expressed sample. Defocus range was set to −0.8 to −1.8 μm. In total, 5002 compressed TIFF movies were acquired at a dose rate of 6.8 e^−^/pixel per s, a pixel size of 0.925 Å, and a cumulative exposure of 50 e^−^/Å^2^. Detailed imaging conditions are described in table S2.

Data from *E. coli*–expressed N(K164A) were collected using the same microscope platform as for the HEK293T cell sample. The nominal defocus range was set to −0.6 to −1.6 μm, and data were acquired in electron event representation (EER) mode. A total of 5733 EER movies were acquired at a dose rate of 9.0 e^−^/pixel per s, a pixel size of 0.724 Å, and a cumulative exposure of 43 e^−^/Å^2^. Detailed imaging conditions are described in table S3.

### Cryo-EM image processing

All datasets were processed following a broadly similar workflow, incorporating motion correction, contrast transfer function (CTF) estimation, iterative 2D classification, and multistep 3D classification to isolate homogeneous subsets before final refinement. Dataset-specific procedures—including particle-picking strategies, box sizes, and classification depths—are described below.

For the *E. coli*–expressed wild-type N dataset, acquired images were chronologically split into six subsets. Gain reference images were generated for each subset using all movie frames with “relion_estimate_gain” command in RELION4-beta (*54*). Subsequent image processing was performed using the software cryoSPARC (*55*, *56*). Motion correction and gain normalization were carried out using “Patch Motion Correction,” and the CTF was estimated using “Patch CTF Estimation.” Particle coordinates were registered with Topaz using the pretrained model ResNet16 (64 units) (*57*), and selected particles were extracted into a 140 × 140–pixel box with a pixel size of 2.64 Å (binning of 4). These particles were subjected to four rounds of 2D classification to remove low-quality particles and to assess and identify diverse, complex structures. Selected particles were then subjected to reference-free 3D reconstruction using “Ab-initio Reconstruction” to generate 3D initial maps. Reference-based 3D classification was performed using “Heterogeneous Refinement” to classify complex structures roughly. A subsequent round of 2D classification was performed to analyze particle population heterogeneity further and to remove remaining contaminants and low-quality particles. Classes containing heterogeneous structures from six subsets were combined and subjected to a subsequent reference-based 3D classification using Heterogeneous Refinement. This step was necessary as most classes still contained a mixture of molecules with varying oligomeric states or conformations. These subsets were categorized as follows: “4-mers”; “5-mer and Flat 6-mer”; 6-mer; “Oval 6-mers”; “7-mer, 8-mer”; and “S-shapes, and Triangles” classes. Resulting subsets were subjected to further 3D classification and particle sorting using multiple rounds of Ab-initio Reconstruction. This step effectively removed remaining low-quality particles and improved class homogeneity. Concurrently, the structural heterogeneity of each complex was visualized and evaluated using “3D Variability Analysis” during 3D classification (movie S1). Final reconstructions were obtained using either “Homogeneous Refinement” or “Non-uniform Refinement.” A detailed image processing workflow is depicted in fig. S1.

For the dataset of HEK293T cell–expressed wild-type N, motion correction and gain normalization using a RELION-made gain reference image were carried out using Patch Motion Correction, and the CTF was estimated using Patch CTF Estimation. Particle coordinates were initially registered with “Blob Picker” to generate 2D templates by subsequent 2D classification for “Template Picker” to enable more accurate particle picking. Picked particles were extracted and subjected to six rounds of 2D classification to remove low-quality particles and to assess and identify complex structures, with an additional round for classes containing multiple tetramers. Because the dataset provided a limited number of particles suitable for high-resolution refinement, stable 3D reconstructions could not be obtained in a reproducible manner. Accordingly, the analysis for this sample was focused on well-resolved 2D class averages.

For the *E. coli*–expressed N(R341A) dataset, motion correction and gain normalization using a gain reference image generated by Thermo Fisher’s software were performed using Patch Motion Correction, and the CTF was estimated using Patch CTF Estimation. Particle coordinates were registered using Template Picker, and selected particles were extracted into a 200 × 200–pixel box with a pixel size of 1.85 Å (binning of 2). These particles were subjected to two rounds of 2D classification to remove low-quality particles and to assess diverse, complex structures. Reference-based 3D classification was performed using Heterogeneous Refinement to classify complex structures roughly into 4-mers, 4-mer antiparallel, and ring-like larger complex classes. After reextracting particles into a rescaled 200 × 200–pixel box with a pixel size of 1.48 Å, two additional rounds of 2D classification were performed for each class to analyze particle population heterogeneity further and to remove remaining contaminants and low-quality particles. Resulting subsets were subjected to further 3D classification and particle sorting using multiple rounds of Ab-initio Reconstruction, yielding classes, namely, 4-mer, asymmetric 4-mer, 4-mer x2 antiparallel, 6-mer, and 7-mer. Final reconstructions were obtained using either Homogeneous Refinement or Non-uniform Refinement. A detailed image processing workflow is depicted in fig. S5E.

For the *E. coli*–expressed N(K164A) dataset, motion correction and gain normalization using a RELION-made gain reference image were carried out using Patch Motion correction, and CTF was estimated using the Patch CTF Estimation. Particle coordinates were registered with Blob Picker, and selected particles were extracted into a 210 × 210–pixel box with a pixel size of 1.448 Å (binning of 2). These particles were subjected to several rounds of 2D classification to remove low-quality particles and to assess and identify diverse, complex structures. Selected 2D classes were used as references, and Template Picker was conducted. Particles were reextracted, classified, and selected in the same manner, and selected particles were then subjected to reference-free 3D reconstruction using Ab-initio Reconstruction to generate 3D initial maps. Reference-based 3D classification was performed using Heterogeneous Refinement to classify complex structures roughly. Another 2D classification was performed to examine what structures were mixed and to remove additional low-quality particles. Structures with high similarity were grouped and subjected to another reference-based 3D classification using Heterogeneous Refinement since most of classes were still a blend of molecules with varying numbers of subunits or conformations, namely, 4-mers, “5-mer and S-shape”, “6-mers”, and “7-mers” classes. Classified structures and particles were subjected to final 3D reconstruction using Homogeneous Refinement and Non-uniform Refinement (only for 4-mers). A detailed image processing workflow is depicted in fig. S8C.

### Atomic model building and refinement

RNA-free N [PDB ID: 1N93 except 9JZJ for N(R341A) 4-mer] was used as the starting model, fitted as a rigid body into cryo-EM maps using UCSF ChimeraX (*58*). The model was then manually adjusted to fit the map with *Coot* (*59*). RNA was modeled de novo as uracil nucleotides using *Coot*. These models were refined with *REFMAC5* (*60*) in *Servalcat* (*61*) using external restraints generated with *ProSMART* (*62*) and *LIBG* (*63*). RNA-free tetramers [wild-type N: 4-mer-1 and 4-mer-2; N(R341A) 4-mer; N(K164A) 4-mer] and RNA-bound hexamer (6-mer) models were refined with C4 and C2 symmetry constraints, respectively, consistent with the symmetry imposed during cryo-EM reconstruction.

### Minireplicon assay

For the minireplicon assay, 1.6 × 10^5^ of HEK293T cells were seeded onto 24-well plates. The following day, cells were transfected with 150 ng of minigenome plasmid having the *Gaussia* luciferase (Gluc) gene as a reporter (*64*), 150 ng of pCAGGS-N (wild-type or mutants), 150 ng of pCAGGS-L, 15 ng of pCAGGS-P, and 35 ng of a control plasmid expressing secreted *Cypridina* luciferase (Cluc) under the control of an SV40 promoter (pSV40-Cluc) using Avalanche-Everyday Transfection Reagent (EZ Biosystems). At 72 hours posttransfection, culture supernatants were subjected to luciferase assays using Pierce Gaussia Luciferase Glow Assay Kit and Pierce Cypridina Luciferase Glow Assay Kit (Thermo Fisher Scientific). Polymerase activity was quantified as the ratio of Gluc activity to Cluc activity, which served as an internal control for transfection efficiency, and expressed as relative light units (RLU), normalized to 1.0 for the negative control lacking expression of L [L (−)].

### Nuclear IB reconstitution assay

U-2 osteosarcoma cells (European Collection of Authenticated Cell Cultures, Public Health England, London, England) were cultured in Dulbecco’s modified Eagle’s medium (Nacalai Tesque Inc., Japan) supplemented with 10% fetal bovine serum. For transfection, cells were seeded on coverslips in 24-well culture plates and cotransfected with 250 ng of plasmid DNA encoding BoDV-1 N and 25 ng of plasmid DNA encoding BoDV-1 P using polyethylenimine “Max” (Polysciences Inc., Warrington, PA, USA). At 24 hours posttransfection, cells were fixed with 4% paraformaldehyde for 10 min, permeabilized, and blocked with 5% bovine serum albumin containing 0.5% Triton X-100 for 15 min. Viral proteins in these cells were then probed with primary antibodies for 2 hours at room temperature (RT), followed by two washes with phosphate-buffered saline (PBS). Subsequently, cells were incubated with secondary antibodies and 4′,6′-diamidino-2-phenylindole (DAPI) for 1 hour at RT, washed three times with PBS, and mounted with ProLong Diamond Antifade Reagent (Life Technologies). Immunofluorescence images were acquired using a laser scanning confocal microscope (LSM 700, Carl Zeiss AG, Switzerland) equipped with a Plan-Apochromat 63× objective lens (numerical aperture = 1.4). The following antibodies were used in this study: anti–BoDV-1 N mouse monoclonal antibody (HN132), anti–BoDV-1 P rabbit polyclonal antibody (HB03), goat anti-mouse IgG (H + L) highly cross-adsorbed secondary antibody conjugated with Alexa Fluor 568 (A-11031, Thermo Fisher Scientific), and goat anti-rabbit IgG (H + L) highly cross-adsorbed secondary antibody conjugated with Alexa Fluor 488 (Thermo Fisher Scientific).

### Data visualization

Molecular visualizations and structural representations were generated using UCSF ChimeraX (version 1.8) (*58*). Plots and graphs were created using gnuplot (version 6.0) (*65*). Multiple sequence alignments of N were performed using MAFFT (*66*) with default parameters. Resulting alignments were visualized and formatted using the ESPript3 server (*67*). All figures were assembled, formatted, and annotated using Adobe Illustrator (version 28.7.1) to ensure consistent style and labeling.

### Statistical analysis

Cryo-EM image processing and model building were performed using standard single-particle analysis methods. 3D reconstruction and resolution estimation were based on the “gold-standard” Fourier shell correlation using the 0.143 criterion, in which two independently refined half-sets of data are compared to minimize reference bias. The quality of final atomic models was assessed using *MolProbity*. The model-to-map fit was evaluated using *Servalcat* software.

Minireplicon assays were performed in technical duplicates across three independent biological replicates. These measurements showed a stark contrast between wild-type and mutant samples. Negative control, L(−), values consistently ranged from 1/90 to 1/170 of wild-type measurements, with mutant samples exhibiting values comparable to those of negative controls. Given the magnitude and consistency of these differences among all replicates, where mutant values were indistinguishable from background levels, the biological significance of observed differences is evident from the raw data, and formal statistical analysis was deemed unnecessary.

Fluorescence microscopy data were obtained from three independent experiments, and representative images are shown. Observed patterns were consistent across all replicates, confirming the reproducibility of these results. To ensure comparability, image acquisition settings were maintained consistently across all samples in each experiment.

## Supplementary Materials

### The PDF file includes:

Figs. S1 to S8 Tables S1 to S4 Legends for movies S1 and S2 References
