# Supplementary material for: Structure and assembly of Borna disease virus 1 nucleoprotein-RNA complexes
Source: Sci Adv. 2026 Apr 10;12(15):eaeb0835. doi: 10.1126/sciadv.aeb0835 (PMC13068073; doi:10.1126/sciadv.aeb0835)
Supplement: Supplementary file 1 — Figs. S1 to S8 Tables S1 to S4 Legends for movies S1 and S2 References [file sciadv.aeb0835_sm.pdf]

Science Advances
AAAS

Supplementary Materials for

Structure and assembly of Borna disease virus 1 nucleoprotein-RNA complexes

Yukihiko Sugita *et al.*

Corresponding author: Yukihiko Sugita, sugita.yukihiko.8w@kyoto-u.ac.jp;
Yuya Hirai, hirai-y@cc.osaka-dent.ac.jp; Masayuki Horie, mhorie@omu.ac.jp

Sci. Adv. **12**, eaeb0835 (2026)
DOI: 10.1126/sciadv.aeb0835

The PDF file includes:

Figs. S1 to S8
Tables S1 to S4
Legends for movies S1 and S2
References

Other Supplementary Material for this manuscript includes the following:

Movies S1 and S2

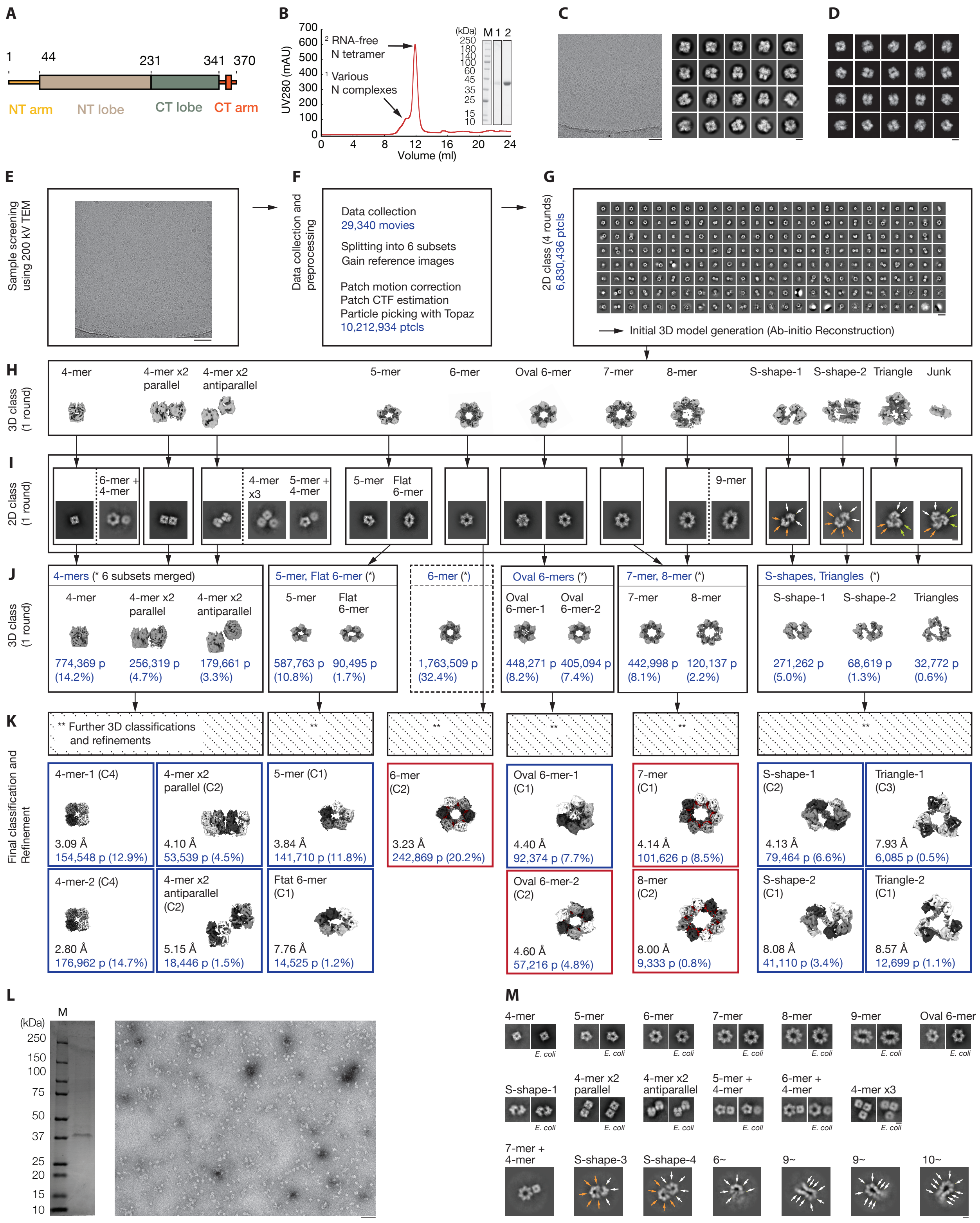

Fig. S1. Expression, purification, and cryo-EM analysis of BoDV-1 N complexes.

(**A**) Schematic of BoDV-1 N domain organization. Bold boxes indicate regions rich in secondary structure. Domain colors for the N-terminal (NT) arm, NT lobe, C-terminal (CT) lobe, and CT arm are consistent across all figures.

(**B**) Size-exclusion chromatography profile of the purified BoDV-1 N complex. Two peaks were fractionated (#1 and #2), with SDS-PAGE analysis shown for each. M: protein marker. These fractions were used for subsequent cryo-EM analyses.

(**C**) Representative cryo-EM image (left) and 2D class averages (right) of complexes from the #2 fraction. Data were acquired on a 200-kV Glacios cryo-TEM and analyzed with RELION4-beta. Scale bars: 50 nm (left) and 50 Å (right).

(**D**) 2D projections of the crystal structure (PDB-ID 1N93) simulated at 9 Å resolution for visual comparison, using the *molmap* command in UCSF Chimera X and the relion_projection command. Scale bar: 50 Å.

(**E** to **K**) Workflow of single-particle cryo-EM for *E. coli*–expressed wild-type N complexes. N complexes from the #1 fraction were vitrified on a grid and examined in the sample screening session using a 200-kV Glacios cryo-TEM, chosen for data collection using a 300-kV microscope. Representative cryo-EM image is shown (E). Scale bar: 50 nm. Following data collection, particles in cryo-EM images were picked, and subjected to 2D classification (G). Scale bar: 20 nm. Selected particles were used to generate 3D reconstructions. These fractions contained a mixture of oligomeric states, including tetramers (4-mers), pentamer (5-mer), hexamers (6-mers), heptamer (7-mer), octamers (8-mer, S-shape-1), nonamers (9-mer, S-shape-2), dodecamers (Triangle-1: 12-mer), and a complex composed of 13 subunits (Triangle-2: 13-mer). Although the nonameric ring-like complex (9-mer) and adjacent oligomers (6-mer + 4-mer, 5-mer + 4-mer, 4-mer x 3) were observed in raw micrographs and 2D class averages, a corresponding 3D reconstruction could not be obtained, due to its sparse representation among the sampled assemblies (I). Scale bar: 50 Å. Classes containing heterogeneous structures from six subsets were combined and subjected to subsequent reference-based 3D classification using "Heterogeneous Refinement." For each grouping, particle numbers used (p) and relative proportions derived from post-classification particle counts are indicated (J). Individual classes were further refined in subsequent classification steps. In total, fifteen cryo-EM maps were reconstructed in the box shown in color at the bottom. In that box, the name of each map (applied symmetry), the overall resolution, and the number of particles used (p) are provided, together with the relative proportions derived from the final particle counts. Blue and red boxes represent RNA-free and RNA-bound complexes, respectively.

(**L**) Expression and purification of BoDV-1 N complexes from HEK293T cells. Left: SDS-PAGE after Ni-NTA affinity purification. M: protein marker. Right: a negative-stain TEM image. Scale bar: 50 nm.

(**M**) Comparison of oligomeric assemblies observed in HEK293T cell–expressed and *E. coli*–expressed N proteins. Top panels: Side-by-side comparison of two-dimensional class averages from HEK293T–cell expression and *E. coli* expression, highlighting tetrameric, pentameric, hexameric, and other assemblies with similar particle diameters in both systems. Bottom panels: Representative two-dimensional class averages showing oligomeric forms detected specifically in the HEK293T cell–derived dataset, including elongated S-shaped assemblies and ring-like or loop-like particles containing nine or more subunits. Scale bar: 50 Å.

In panels I and M (bottom), subunits that can be individually counted in the 2D class averages are indicated by arrows: orange and green arrows mark the terminal subunits in S-shaped assemblies and the vertex subunits of triangular complexes, respectively, while white arrows denote identifiable subunits in loop-like assemblies.

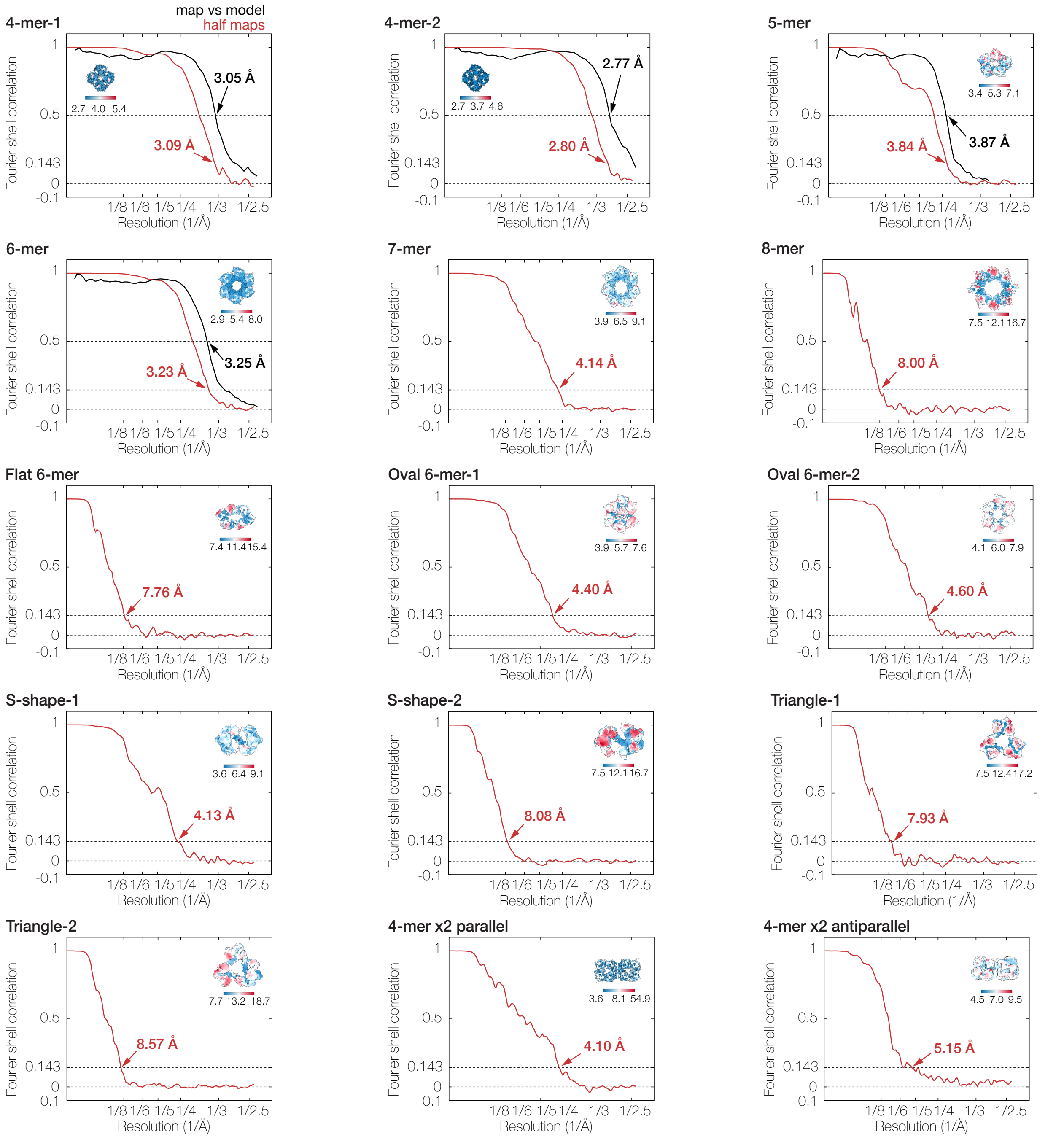

Fig. S2. Resolution estimation plots for cryo-EM reconstructions of *E. coli*–expressed wild-type N complexes.

Resolution estimation plots for cryo-EM reconstruction. Red curve: gold-standard FSC between independent half-maps (threshold at FSC = 0.143). Black curve: FSC between the full cryo-EM map and the atomic model (map vs model, threshold at FSC = 0.5). Local resolution maps are shown with color scales from blue to red (Å).

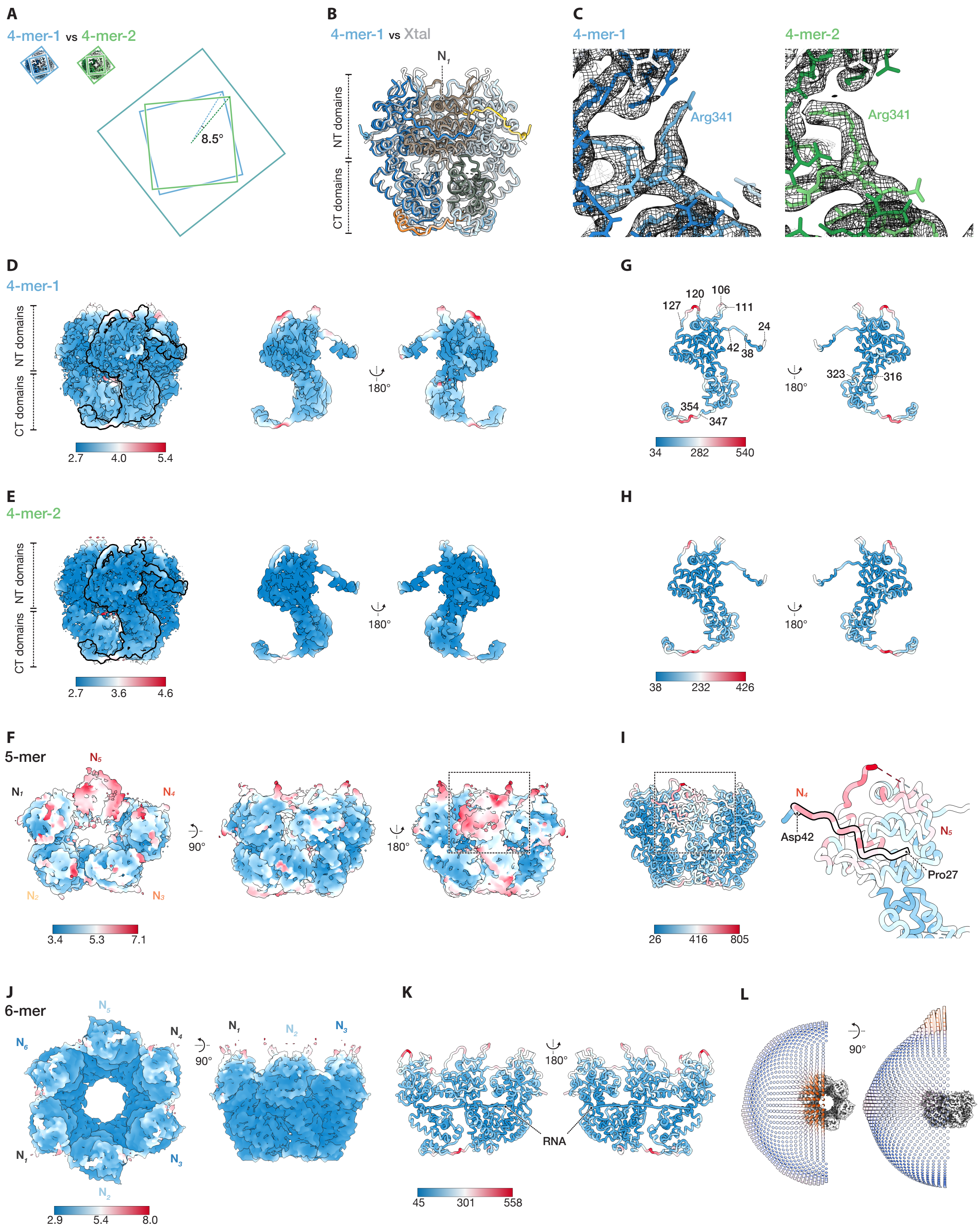

Fig. S3. Structural comparisons and local resolution of cryo-EM maps.

(**A**) Schematic illustrating the angular difference between C-terminal and N-terminal lobes of 4-mer-1 (blue) and 4-mer-2 (green). Bottom views showing N-terminal (large square) and C-terminal lobe (small square) are presented at top left.

(**B**) Structural alignment of 4-mer-1 (colored) with crystal structure (Xtal, transparent grey). Atomic models are shown as Cα backbone wire representations. The crystal structure exhibits a more compact conformation.

(**C**) Close-up view of the local cryo-EM density around Arg341. The density map is shown in mesh representation with the fitted atomic model superimposed, highlighting the side-chain placement and the inter-subunit interface. This zoomed-in comparison illustrates the conformational environment of Arg341 observed in the 4-mer-1 and 4-mer-2 structures.

(**D** to **F**) Local resolution maps of cryo-EM reconstructions for 4-mer-1 (D), 4-mer-2 (E), and 5-mer complexes (F). For tetramers: complete complex maps (left panel) and segmented asymmetric subunit maps (right panels). Color scale ranges from blue to red (Å), as indicated by the color bar.

(**G** to **I**) Atomic models colored by *B*-factor for 4-mer-1 (G), 4-mer-2 (H), and 5-mer (I). The color scale ranges from blue (low *B*-factor, indicating more stable regions) to red (high *B*-factor, indicating more flexible regions) in $Å^2$, as indicated by the color bar. In (I), a close-up view highlights the region with the highest *B*-factors (dotted boxes); amino acid numbers of the N-terminal arm are shown in the right panel.

(**J** and **K**) 6-mer complex: Local resolution map (J) and atomic model (K) colored by *B*-factor. Color scales are the same as in panels (D to I).

(**L**) Angular distribution of the 6-mer reconstruction shown as a 3D histogram. With C2 symmetry imposed, orientations are displayed over a hemisphere. This distribution reflects substantial angular coverage, including both top and side views, indicating that the reconstruction is supported by a broad range of particle orientations.

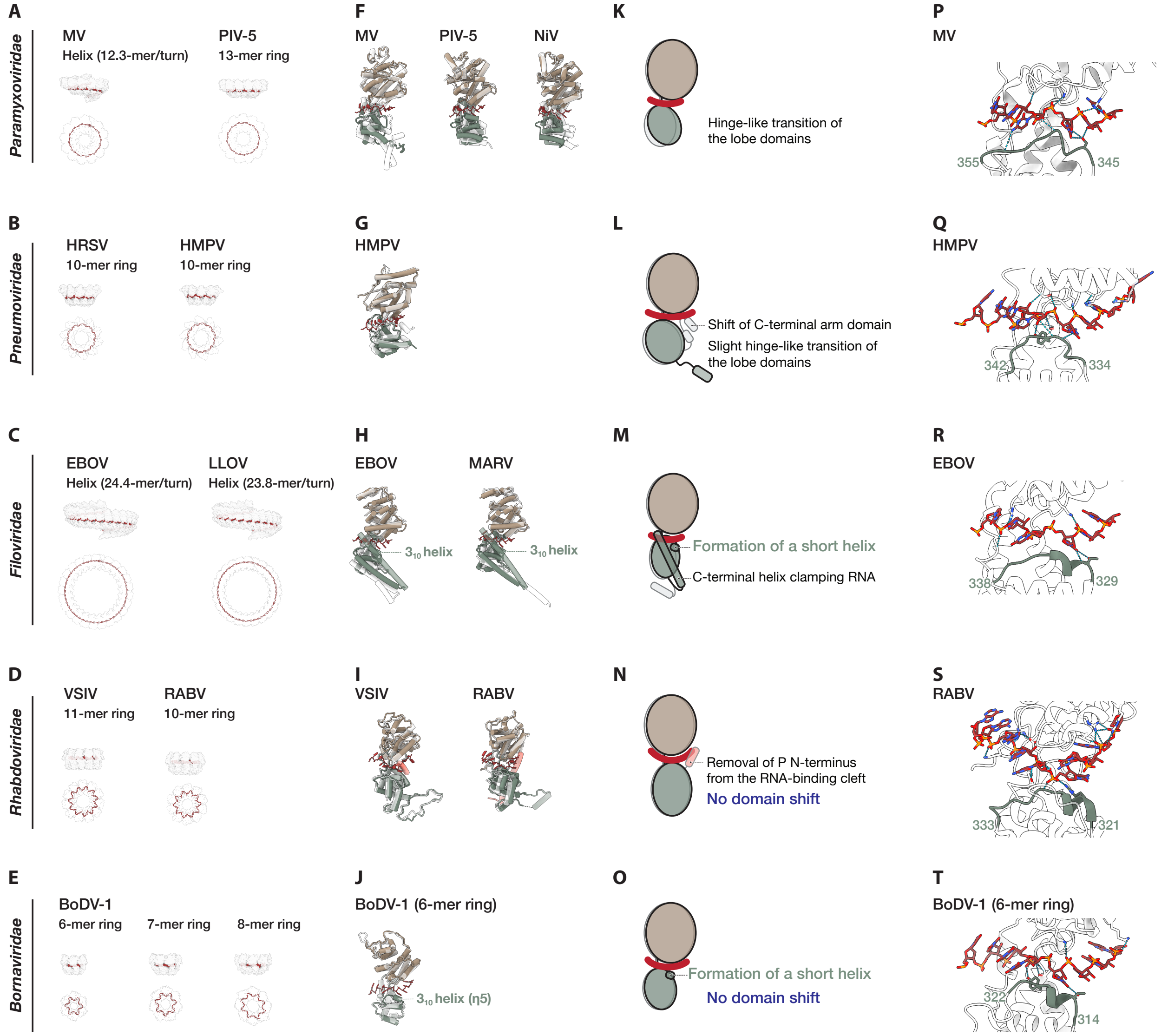

Fig. S4. RNA structures in viral N–RNA complexes in the order *Mononegavirales*.

(**A** to **E**) Atomic models of representative N–RNA complexes from five families in the *Mononegavirales*, shown in surface representation. RNA is depicted in red using a ladder representation in the top view to highlight the configuration of the RNA backbone and base arrangement: measles virus (MV, PDB-ID 4UFT (*11*)) and parainfluenza virus 5 (PIV5, PDB-ID 4XJN (*30*)), *Paramyxoviridae* (A); Human respiratory syncytial virus (HRSV, PDB-ID 2WJ8 (*10*)) and human metapneumovirus (HMPV, PDB-ID 5FVC (*15*)), *Pneumoviridae* (B); Ebola virus (EBOV, PDB-ID 5Z9W (*16*)) and Lloviu virus (LLOV, PDB-ID 7YPW (*21*)), *Filoviridae* (C); vesicular stomatitis Indiana virus (VSIV, PDB-ID 2GIC (*7*)) and Rabies virus (RABV, PDB-ID 2GTT (*8*)), *Rhabdoviridae* (D); BoDV-1 (6-mer, PDB-ID 9JZI, this study), *Bornaviridae* (E). For the heptameric and octameric N–RNA complex, the NT-arm, core, and CT-arm of a subunit in the hexameric model are independently fitted into cryo-EM maps as rigid bodies.

(**F** to **J**) Conformational shift and RNA-binding motifs of N molecules among members of the order *Mononegavirales*. In each panel, the RNA-bound state (N–RNA, color) is overlaid with the

RNA-free state (N^0, grey) to facilitate structural comparison. MV (N–RNA, PDB-ID 4UFT; N^0-P, PDB-ID 5E4V (*12*)), PIV-5 (N–RNA, PDB-ID 4XJN; N^0-P, PDB-ID 5WKN (*68*)) and Nipah virus (NiV; N–RNA, PDB-ID 7NT5 (*24*); N^0-P, PDB-ID 4CO6 (*69*)), *Paramyxoviridae* (G). HMPV (N–RNA, PDB-ID 5FVC (*15*); N^0-P, PDB-ID 5FVD (*15*)), *Pneumoviridae* (H). EBOV (NP–RNA, PDB-ID 5Z9W (*16*); NP^0-VP35, PDB-ID 4YPI (*14*)) and Marburg virus (MARV; NP–RNA, PDB-ID 7F1M (*20*); NP^0-VP35, PDB-ID 5F5M), *Filoviridae* (I). VSIV (N–RNA, PDB-ID 2GIC; N^0-P, PDB-ID 3PMK (*70*)) and RABV (N–RNA, PDB-ID 8FFR (*17*); N^0-P, PDB-ID 8B8V (*17*)), *Rhabdoviridae* (J). BoDV-1 (N–RNA, PDB-ID 9JZL, this study; N^0-P, PDB-ID 1N93 (*6*)), *Bornaviridae* (K). Note: In filoviruses, nucleoprotein and phosphoprotein are conventionally abbreviated to NP and VP35, respectively.

(**K** to **O**) Schematic diagrams corresponding to panels (G to K), with annotated text highlighting key structural differences between RNA-free and RNA-bound states for each virus.

(**P** to **T**) Close-up of N–RNA binding interface for: MV (P), HMPV (Q), EBOV (R), RABV (S), and BoDV-1 (T). Blue dashed lines indicate residues within hydrogen bond distance. RNA and RNA-binding loops are colored and labeled, whereas other regions of the protein are rendered in translucent white for clarity.

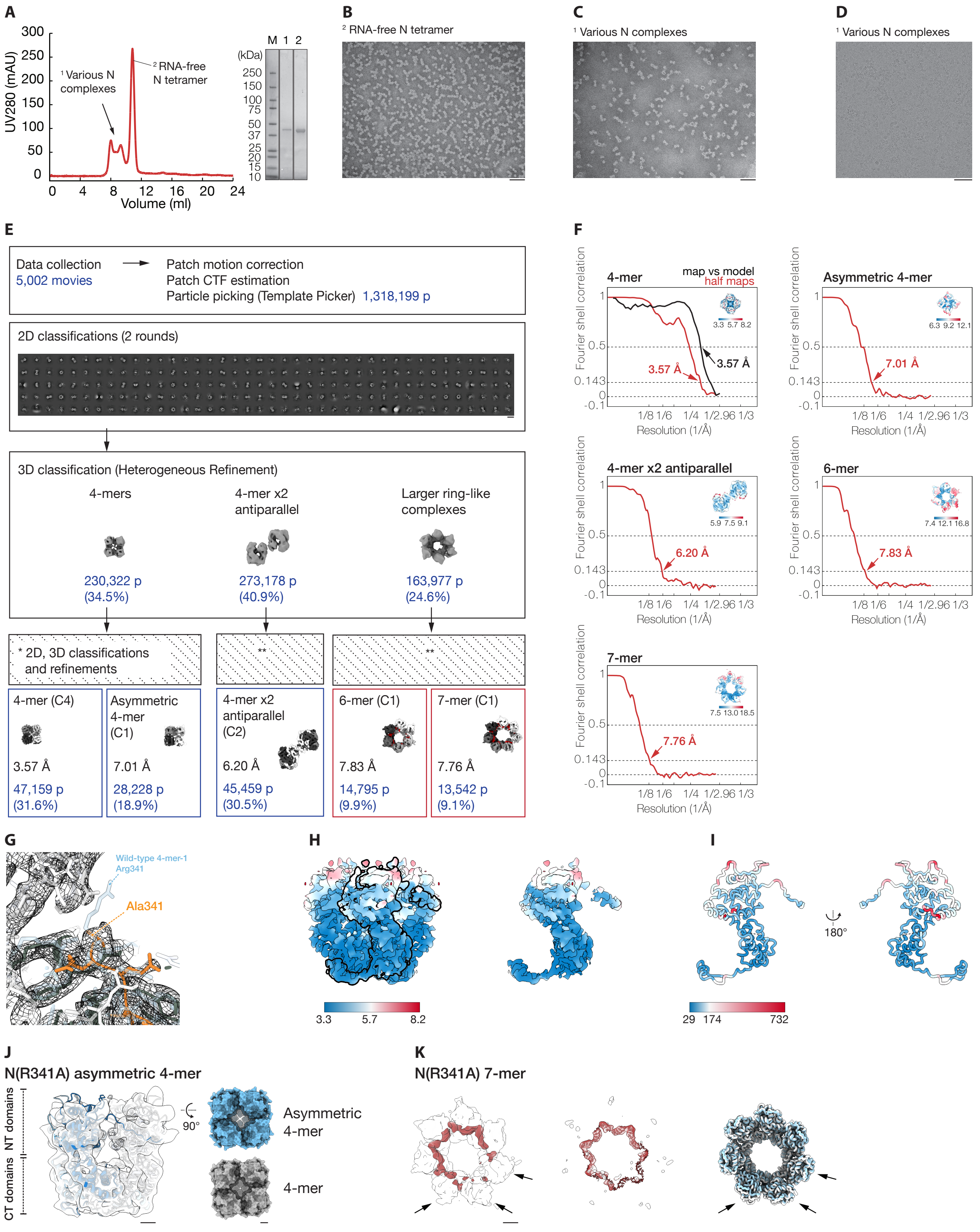

Fig. S5. Expression, purification, and cryo-EM analysis of BoDV-1 N(R341A) complexes.

(**A**) Size-exclusion chromatography profile of the purified BoDV-1 N(R341A) complex. Two peaks were fractionated (#1 for various complexes and #2 for RNA-free tetramer), with SDS-PAGE analysis shown for each. These fractions were used for subsequent negative-stain TEM and cryo-EM analyses. M: protein marker.

(**B** and **C**) Representative negative-stain TEM images of fraction #2 (B) and #1 (C), showing small clusters of particles. Scale bars: 50 nm (left).

(**D**) Representative cryo-EM image of fraction #1. Scale bar: 50 nm.

(**E**) Workflow of single-particle cryo-EM for N(R341A) complexes in #1 fractions. Particles in cryo-EM images were picked using 2D templates and classified in 2D. Scale bar: 20 nm. 2D classes show a mixture of oligomeric states, including tetramers (4-mers), adjacent 4-mers (4-mer x2 antiparallel), and larger ring-like complexes containing hexamers and heptamers. A rough 3D classification was performed using "Heterogeneous Refinement." Five cryo-EM maps were reconstructed in the box shown in color at the bottom. In that box, the name of each map (applied symmetry), overall resolution, the number of particles used (p), and the relative proportions of particle counts are indicated. Blue and red boxes represent RNA-free and RNA-bound complexes, respectively.

(**F**) Resolution estimation plots for cryo-EM reconstruction. Red curve: gold-standard FSC between independent half-maps (threshold at FSC = 0.143). Black curve: FSC between the full cryo-EM map and the atomic model (threshold at FSC = 0.5). Local resolution maps are shown in each panel, with color scales ranging from blue to red in Å, as indicated by the bar.

(G) Close-up view of the local cryo-EM density of the N(R341A) 4-mer around the Arg341Ala mutation site. The density map is shown in mesh representation, with fitted atomic models superimposed, showing that the local backbone environment remains highly similar between the N(R341A) 4-mer (colored by domains, with adjacent subunits in grey) and the wild-type 4-mer-1 (transparent light blue) despite the absence of the Arg341 side chain.

(**H**) Local resolution of complete 4-mer complex maps (left panel) and segmented asymmetric subunit maps (right panel). Color scale ranges from blue to red (Å), as indicated by the color bar.

(**I**) Atomic models colored by *B*-factor for 4-mer. The color scale is the same as in fig. S3, illustrating enhanced fluctuation in the N-terminal domains.

(**J**) Left panel: Asymmetric 4-mer map superposed with rigid-body–fitted 4-mer-1 subunits in ribbon representation, showing a reasonable fit of the model, particularly for the C-terminal domain, in the map. Right panels: Surface representations of the asymmetric model (blue) and the 4-mer model (grey), showing an enlarged channel between the N-terminal domains. Scale bars: 10 Å.

(**K**) Left: the full cryo-EM map of the N(R341A) 7-mer. The density colored in red was obtained from a difference map generated by subtracting a 7.83 Å resolution map calculated from the fitted model of the wild-type 6-mer (chains A and R, multiplied seven times) using the *molmap* function in UCSF ChimeraX from the N(R341A) 7-mer map. This density is located near the RNA-binding cleft. The difference density contains a particularly weak signal corresponding to RNA associated with only three adjacent subunits (arrows). Middle: the difference map, with the RNA model shown in stick representation. Right: superposition of the core domains, shown in wire representation, of the rigid-body–fitted N(R341A) 7-mer protein model with the wild-type 7-mer model, generated by fitting chains A and R multiplied seven times into the wild-type 7-

mer map, showing substantial deviations in the spatial arrangement of two to three subunits (arrows). Scale bars: 20 Å.

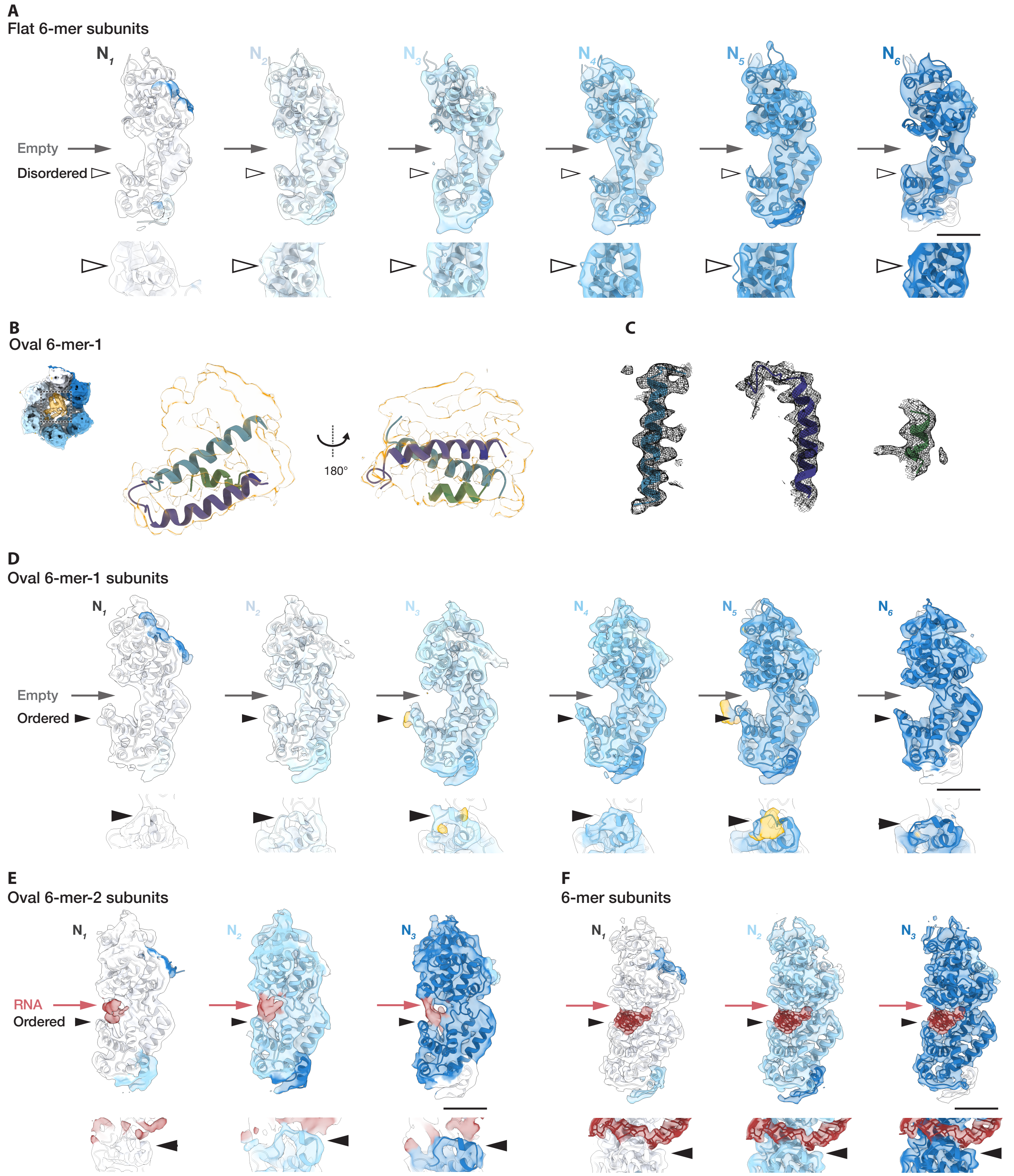

Fig. S6. Detailed structural analysis for elongated hexameric complexes.

(**A**) Isolated cryo-EM maps of asymmetric N subunits of Flat 6-mer, superimposed with atomic models in ribbon representation.

(**B**) Isolated central cryo-EM density in Oval 6-mer-1 complex with assignments of α-helices in a ribbon representation predicted by ModelAngelo.

(**C**) Isolated cryo-EM density in a mesh representation for α-helices illustrating a good match for the helical pattern of grooves.

(**D** to **F**) Isolated cryo-EM maps of asymmetric N subunits, superimposed with atomic models in ribbon representation: Oval 6-mer-1 (D), Oval 6-mer-2 (E), and RNA-bound 6-mer (reference for RNA cryo-EM density) (F). Atomic models of the tetramer (4-mer-2) for Flat 6-mer and 6-mer for oval 6-mers were initially fit as a rigid body, and then flexibly fit using molecular dynamics flexible fitting (MDFF) as implemented in ISOLDE (*71*), within UCSF ChimeraX.

Scale bars: 20 Å.

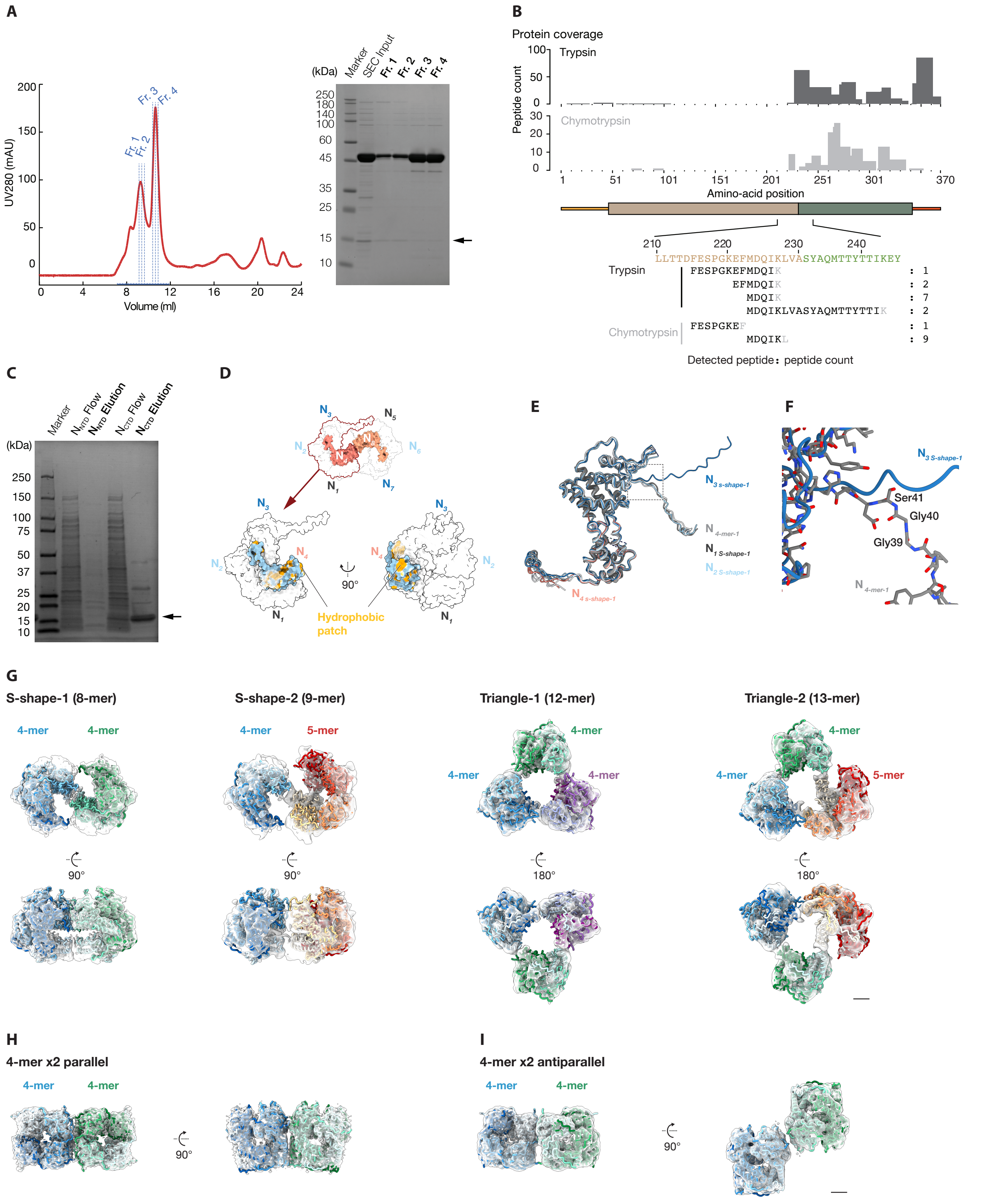

Fig. S7. Complex assemblies and unique protein-protein interactions involving N_{CTD}.

(**A**) Purification and characterization of BoDV-1 N protein. Left: SEC profile of purified BoDV-1 N protein. Blue dashed lines indicate fractions for further analysis. Right: SDS-PAGE of SEC

fractions. A band distinct from full-length N (arrow) was excised from Fraction #2 and analyzed by LC-MS/MS.

(**B**) LC-MS/MS analysis of BoDV-1 N protein (Q Exactive Plus, Thermo Fisher Scientific). Samples were digested with trypsin or chymotrypsin. Top: Histogram showing peptide coverage across the N protein sequence. Bottom: Detected amino-acid sequences and their counts in the boundary region between the C-terminal and N-terminal lobes. Residues presumed to have been cleaved at the carboxy terminus by protease are highlighted in grey. Putative CTD cleavage sites were identified at positions 214D-215F, 220K-221E, and 222F-223M.

(**C**) SDS-PAGE analysis of the recombinantly expressed and purified N_{NTD} (residues 1 to 230) and N_{CTD} (residues 231 to 370), from Ni-NTA affinity purification. Lanes (from left to right): Marker, protein marker; flow-through fraction of N_{NTD} (showing comparable bacterial loading across samples); elution peak of N_{NTD}; flow-through fraction of N_{CTD}; elution peak of N_{CTD}, showing a low-molecular-weight band similar to the small fragment observed in full-length N preparations (arrow).

(**D**) Binding interface between the cleaved N_{CTD} in the S-shape-1 complex. Top: Surface representation of the S-shape-1 complex, with bridging subunits N_4 and N_8 highlighted in pink. The red outline indicates the area enlarged in the lower panels. Bottom: hydrophobic/hydrophilic surface potential of the outlined region, calculated using the Brasseur method (*66*), colored cyan (hydrophilic) and orange (hydrophobic).

(**E** and **F**) Structural comparison of subunits from the S-shape-1 and 4-mer-1. N-terminal arm in the S-shape-1 complex exhibits a sharp bend (E), centered at a Gly-Gly-Ser motif (residues 39-41) (F).

(**G**) Spatial arrangement of subunits in the S-shaped, triangular complexes. Atomic models of tetrameric complexes (cool colors) and pentameric complexes (warm colors) are superimposed to show well-aligned architecture. Scale bar: 20 Å.

(**H** and **I**) Tetrameric complexes aligned in parallel (H) and antiparallel configurations (I). Scale bar: 20 Å.

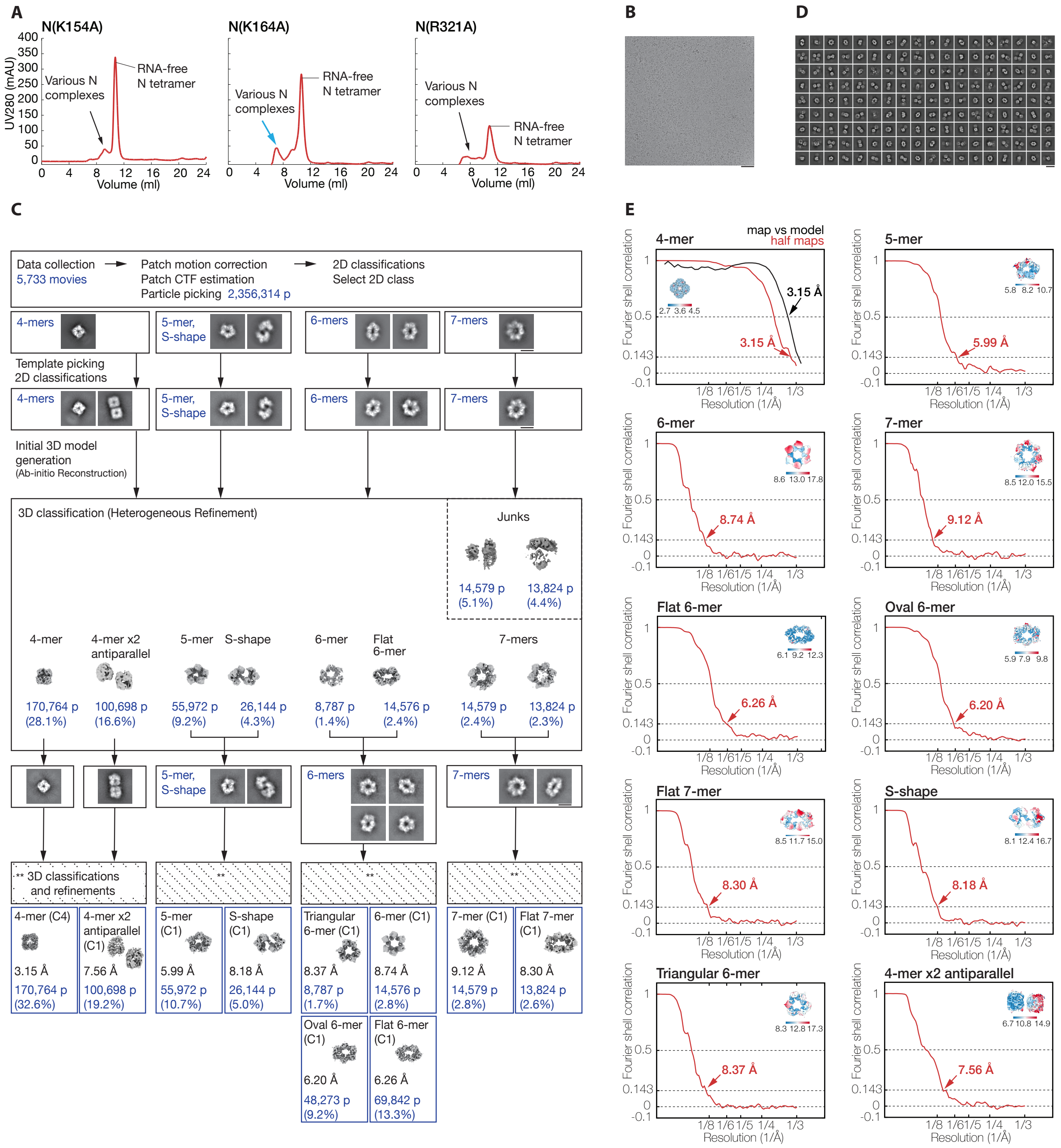

Fig. S8. Purification and structural analysis of N mutants

(**A**) SEC profiles for N mutants: N(K154A), N(K164A), and N(R321A). Arrows indicate fractionation zones, with a cyan arrow marking the fraction subjected to cryo-EM analysis.

(**B**) Representative cryo-EM image of purified N(K164A) complexes from fractions that eluted earlier. Scale bar: 50 nm.

(**C**) Workflow of the single-particle cryo-EM performed for N(K164A) complexes. Scale bars: 20 nm.

(**D**) Representative 2D class averages of N(K164A), demonstrating structural heterogeneity and pleomorphism. Scale bar: 20 nm.

(**E**) Resolution estimation plots for cryo-EM reconstruction. Red curve: gold-standard FSC between independent half-maps (threshold at FSC = 0.143). Black curve: FSC between the full cryo-EM map and the atomic model (threshold at FSC = 0.5). Local resolution maps are shown in each panel, with color scales ranging from blue to red in Å, as indicated by the bar.

Table S1. Cryo-EM analysis and atomic model building for wild-type N

	4-mer-1 (EMD-61914) (PDB-ID 9JZI)	4-mer-2 (EMD-61915) (PDB-ID 9JZJ)	5-mer (EMD-61916) (PDB-ID 9JZK)	6-mer (EMD-61917) (PDB-ID 9JZL)
**Data collection and image processing**				
Magnification			81,000	
Voltage (kV)			300	
Detector			K3 BioQuantum	
Energy filter (eV)			20	
Total electron exposure ($e^{-}/Å^2$)			40	
Electron dose rate ($e^{-}/Å^2/s$)			7	
Dose fractionation (frames/sec)			5	
Defocus range (μm)			-0.6 to -1.6	
Pixel size (Å)			0.88	
Total image (no.)			29,340	
Final particles (no.)	154,548	176,962	141,710	242,869
Symmetry imposed	C4	C4	C1	C2
Map resolution (Å)	3.09	2.80	3.84	3.23
FSC threshold	0.143	0.143	0.143	0.143
Map sharpening B factor ($Å^2$)	118.4	108.3	128.3	70.0
**Refinement**				
Initial model used (PDB ID)	1N93	1N93	1N93	1N93
Model resolution (Å)	3.05	2.78	3.85	3.21
FSC threshold	0.5	0.5	0.5	0.5
Model composition in the asymmetric unit				
Protein residues	337 (1 chain)	337 (1 chain)	1,622 (5 chains)	1,022 (3 chains)
nucleotides	-	-	-	24 (1 chain)
Non-hydrogen atoms	2,616 (1 chain)	2,616 (1 chain)	12,633 (5 chains)	8,422 (3 chains)
B factor ($Å^2$)	97.8	82.1	208.7	122.6
R.m.s. deviations				
Bond lengths (Å)	0.0111	0.0119	0.0114	0.0110
Bond angles (°)	1.65	1.61	1.85	1.61
Validation				
MolProbity score	0.89	0.98	1.54	1.26
Clashscore	1.52	2.09	4.97	2.79
Poor rotamers (%)	0.35	0.00	1.10	0.12
Ramachandran plot				
Favored (%)	98.49	98.49	96.29	96.83
Allowed (%)	1.51	1.51	3.71	3.17
Disallowed (%)	0.00	0.00	0.00	0.00

	7-mer (EMD-61918)	8-mer (EMD-61919)	Flat 6-mer (EMD-61920)	Oval 6-mer-1 (EMD-61921)	Oval 6-mer-2 (EMD-61922)	S-shape-1 (EMD-61923)
**Data collection and image processing**						
Magnification				81,000		
Voltage (kV)				300		
Detector				K3 BioQuantum		
Energy filter (eV)				20		
Total electron exposure ($e^{-}/Å^2$)				40		
Electron dose rate ($e^{-}/Å^2/s$)				7		
Dose fractionation (frames/sec)				5		
Defocus range (μm)				-0.6 to -1.6		
Pixel size (Å)				0.88		
Total image (no.)				29,340		
Final particles (no.)	101,626	9,333	14,525	92,374	57,216	79,464
Symmetry imposed	C1	C2	C1	C1	C2	C2
Map resolution (Å)	4.14	8.00	7.76	4.40	4.60	4.13
FSC threshold	0.143	0.143	0.143	0.143	0.143	0.143
Map sharpening *B* factor ($Å^2$)	122.2	638.8	511.4	158.1	195.4	100.0

	S-shape-2 (EMD-61924)	Triangle-1 (EMD-61925)	Triangle-2 (EMD-61926)	4-mer x2 parallel (EMD-61927)	4-mer x2 antiparallel (EMD-61928)
**Data collection and image processing**					
Magnification			81,000		
Voltage (kV)			300		
Detector			K3 BioQuantum		
Energy filter (eV)			20		
Total electron exposure ($e^{-}/Å^2$)			40		
Electron dose rate ($e^{-}/Å^2/s$)			7		
Dose fractionation (frames/sec)			5		
Defocus range (μm)			-0.6 to -1.6		
Pixel size (Å)			0.88		
Total image (no.)			29,340		
Final particles (no.)	41,110	6,085	12,699	53,539	18,446
Symmetry imposed	C1	C3	C1	C2	C2
Map resolution (Å)	8.08	7.93	8.57	4.10	5.15
FSC threshold	0.143	0.143	0.143	0.143	0.143
Map sharpening *B* factor ($Å^2$)	300.0	400.0	400.0	69.8	135.1

Table S2. Cryo-EM analysis and atomic model building for N(R341A)

	4-mer (EMD-67470) (PDB-ID 21AL)	Asymmetric 4-mer (EMD-67471)	4-mer x2 antiparallel (EMD-67472)	6-mer (EMD-67473)	7-mer (EMD-67474)
**Data collection and image processing**					
Magnification			150,000		
Voltage (kV)			200		
Detector			Falcon4		
Energy filter (eV)			none		
Total electron exposure (e^{-}/Å^{2})			50		
Electron dose rate (e^{-}/Å^{2}/s)			8.0		
Dose fractionation (frames/sec)			10.6		
Defocus range (μm)			-0.8 to -1.8		
Pixel size (Å)			0.925		
Total image (no.)			5,002		
Final particles (no.)	47,159	28,228	45,459	14,795	13,542
Symmetry imposed	C4	C1	C2	C1	C1
Map resolution (Å)	3.57	7.01	6.20	7.83	7.76
FSC threshold	0.143	0.143	0.143	0.143	0.143
Map sharpening *B* factor (Å^{2})	100.0	300.0	475.0	523.5	486.2
**Refinement**					
Initial model used (PDB ID)	9JZJ				
Model resolution (Å)	3.57				
FSC threshold	0.5				
Model composition in the asymmetric unit					
Protein residues	328 (1 chain)				
nucleotides	-				
Non-hydrogen atoms	2591 (1 chain)				
B factor (Å^{2})	173.9				
R.m.s. deviations					
Bond lengths (Å)	0.0107				
Bond angles (°)	1.84				
Validation					
MolProbity score	1.68				
Clashscore	5.62				
Poor rotamers (%)	0.35				
Ramachandran plot					
Favored (%)	94.51				
Allowed (%)	5.18				
Disallowed (%)	0.30				

Table S3. Cryo-EM analysis and atomic model building for N(K164A)

	4-mer (EMD-61929) (PDB-ID 9JZN)	5-mer (EMD-61930)	6-mer (EMD-61931)	7-mer (EMD-61932)
**Data collection and image processing**				
Magnification	190,000			
Voltage (kV)	200			
Detector	Falcon4			
Energy filter (eV)	none			
Total electron exposure (e^{-}/Å^2)	43			
Electron dose rate (e^{-}/Å^2/s)	9.2			
Dose fractionation (frames/sec)	8.5			
Defocus range (µm)	-0.6 to -1.6			
Pixel size (Å)	0.724			
Total image (no.)	5,733			
Final particles (no.)	170,764	55,972	14,576	14,579
Symmetry imposed	C4	C1	C1	C1
Map resolution (Å)	3.15	5.99	8.74	9.12
FSC threshold	0.143	0.143	0.143	0.143
Map sharpening *B* factor (Å^2)	110.0	409.7	911.1	914.4
**Refinement**				
Initial model used (PDB ID)	1N93			
Model resolution (Å)	3.19			
FSC threshold	0.5			
Model composition in the asymmetric unit				
Protein residues	336 (1 chain)			
nucleotides	-			
Non-hydrogen atoms	2607 (1 chain)			
B factor (Å^2)	132.1			
R.m.s. deviations				
Bond lengths (Å)	0.0118			
Bond angles (°)	1.67			
Validation				
MolProbity score	1.59			
Clashscore	4.06			
Poor rotamers (%)	0.35			
Ramachandran plot				
Favored (%)	95.15			
Allowed (%)	4.85			
Disallowed (%)	0.00			

	Flat 6-mer (EMD-61933)	Oval 6-mer (EMD-61934)	Flat 7-mer (EMD-61935)	S-shape (EMD-61936)	Triangular 6-mer (EMD-61937)	4-mer x2 antiparallel (EMD-61938)
**Data collection and image processing**						
Magnification	190,000					
Voltage (kV)	200					
Detector	Falcon4					
Energy filter (eV)	none					
Total electron exposure (e^{-}/Å^2)	40					
Electron dose rate (e^{-}/Å^2/s)	7					
Dose fractionation (frames/sec)	5					
Defocus range (µm)	-0.6 to -1.6					
Pixel size (Å)	0.724					
Total image (no.)	5,733					
Final particles (no.)	69,842	48,273	13,824	26,144	8,787	100,698
Symmetry imposed	C1	C1	C1	C1	C1	C1
Map resolution (Å)	6.26	6.20	8.30	8.18	8.37	7.56
FSC threshold	0.143	0.143	0.143	0.143	0.143	0.143
Map sharpening *B* factor (Å^2)	464.7	402.0	655.1	618.7	539.3	746.3

Table S4. Comparative structural features of N–RNA complexes among families in the order *Mononegavirales*

	**Human virus families in the order *Mononegavirales***				
	Bornaviridae	***Rhabdoviridae***	***Pneumoviridae***	***Paramyxoviridae***	***Filoviridae***
RNA location in the complex	Inside	Inside	Outside	Outside	Outside
Number of nucleotides per nucleoprotein	8	9	7	6	6
Orientation of RNA bases	4-in, 4-out	3-in, 6-out	3-in, 4-out	3-in, 3-out	3-in, 3-out
N–N interactions *	NT-arm<br>CT-arm	NT-arm<br>CT-extended loop	NT-arm<br>CT-arm	NT-arm<br>CT-arm	NT-arm<br>CT-helix

* NT: N-terminal, CT: C-terminal

movie S1. Visualization of continuous conformational heterogeneity in BoDV-1 N complexes

3D Variability Analysis in CryoSPARC revealed the continuous conformational landscape of N complexes. Animation cycles through seven distinct assemblies of N complexes. This approach complemented the distinct assembly states identified by 3D classification, revealing structural heterogeneity that suggests continuous conformational changes in each state. Scale bar: 10 Å.

movie S2. An assembly model of the hexameric ring-like N–RNA complex

This morph movie illustrates a possible assembly pathway of the BoDV-1 N–RNA complex, starting from an RNA-free tetramer and sequentially transitioning through a twin tetramer, an S-shaped complex, a flat hexamer, an oval hexamer, and finally a fully assembled ring-like hexamer bound to RNA. Dynamic transformations highlight the structural plasticity of the N protein and suggest potential intermediates in nucleocapsid assembly, providing insights into possible stepwise assembly of the functional N–RNA complex.

REFERENCES

1. J. H. Kuhn, S. Adkins, S. V. Alkhovsky, T. Avšič-Županc, M. A. Ayllón, J. Bahl, A. Balkema-Buschmann, M. J. Ballinger, M. Bandte, M. Beer, N. Bejerman, É. Bergeron, N. Biedenkopf, L. Bigarré, C. D. Blair, K. R. Blasdell, S. B. Bradfute, T. Briese, P. A. Brown, R. Bruggmann, U. J. Buchholz, M. J. Buchmeier, A. Bukreyev, F. Burt, C. Büttner, C. H. Calisher, T. Candresse, J. Carson, I. Casas, K. Chandran, R. N. Charrel, Y. Chiaki, A. Crane, M. Crane, L. Dacheux, E. D. Bó, J. C. De La Torre, X. De Lamballerie, W. M. De Souza, R. L. De Swart, N. M. Dheilly, N. Di Paola, F. Di Serio, R. G. Dietzgen, M. Digiaro, J. F. Drexler, W. P. Duprex, R. Dürrwald, A. J. Easton, T. Elbeaino, K. Ergünay, G. Feng, C. Feuvrier, A. E. Firth, A. R. Fooks, P. B. H. Formenty, J. Freitas-Astúa, S. Gago-Zachert, M. L. García, A. García-Sastre, A. R. Garrison, S. E. Godwin, J.-P. J. Gonzalez, J. G. De Bellocq, A. Griffiths, M. H. Groschup, S. Günther, J. Hammond, J. Hepojoki, M. M. Hierweger, S. Hongō, M. Horie, H. Horikawa, H. R. Hughes, A. J. Hume, T. H. Hyndman, D. Jiāng, G. B. Jonson, S. Junglen, F. Kadono, D. G. Karlin, B. Klempa, J. Klingström, M. C. Koch, H. Kondō, E. V. Koonin, J. Krásová, M. Krupovic, K. Kubota, I. V. Kuzmin, L. Laenen, A. J. Lambert, J. Lǐ, J.-M. Li, F. Lieffrig, I. S. Lukashevich, D. Luo, P. Maes, M. Marklewitz, S. H. Marshall, S.-Y. L. Marzano, J. W. McCauley, A. Mirazimi, P. G. Mohr, N. J. G. Moody, Y. Morita, R. N. Morrison, E. Mühlberger, R. Naidu, T. Natsuaki, J. A. Navarro, Y. Neriya, S. V. Netesov, G. Neumann, N. Nowotny, F. M. Ochoa-Corona, G. Palacios, L. Pallandre, V. Pallás, A. Papa, S. Paraskevopoulou, C. R. Parrish, A. Pauvolid-Corrêa, J. T. Paweska, D. R. Pérez, F. Pfaff, R. K. Plemper, T. S. Postler, F. Pozet, S. R. Radoshitzky, P. L. Ramos-González, M. Rehanek, R. O. Resende, C. A. Reyes, V. Romanowski, D. Rubbenstroth, L. Rubino, A. Rumbou, J. A. Runstadler, M. Rupp, S. Sabanadzovic, T. Sasaya, H. Schmidt-Posthaus, M. Schwemmle, T. Seuberlich, S. R. Sharpe, M. Shi, M. Sironi, S. Smither, J.-W. Song, K. M. Spann, J. R. Spengler, M. D. Stenglein, A. Takada, R. B. Tesh, J. Těšíková, N. J. Thornburg, N. D. Tischler, Y. Tomitaka, K. Tomonaga, N. Tordo, K. Tsunekawa, M. Turina, I. E. Tzanetakis, A. M. Vaira, B. Van Den Hoogen, B. Vanmechelen, N. Vasilakis, M. Verbeek, S. Von Bargen, J. Wada, V. Wahl, P. J. Walker, A. E. Whitfield, J. V. Williams, Y. I. Wolf, J. Yamasaki, H. Yanagisawa, G. Ye, Y.-Z. Zhang, A. L. Økland, 2022 taxonomic update of phylum Negarnaviricota (Riboviria: Orthornavirae), including the large orders Bunyavirales and Mononegavirales. *Arch. Virol.* **167**, 2857–2906 (2022).

2. X. Lahaye, A. Vidy, C. Pomier, L. Obiang, F. Harper, Y. Gaudin, D. Blondel, Functional characterization of Negri bodies (NBs) in rabies virus-infected cells: Evidence that NBs are sites of viral transcription and replication. *J. Virol.* **83**, 7948–7958 (2009).

3. T. Hoenen, R. S. Shabman, A. Groseth, A. Herwig, M. Weber, G. Schudt, O. Dolnik, C. F. Basler, S. Becker, H. Feldmann, Inclusion bodies are a site of ebolavirus replication. *J. Virol.* **86**, 11779–11788 (2012).

4. N. Cifuentes-Muñoz, J. Branttie, K. B. Slaughter, R. E. Dutch, Human metapneumovirus induces formation of inclusion bodies for efficient genome replication and transcription. *J. Virol.* **91**, e01282-17 (2017).

5. V. Rincheval, M. Lelek, E. Gault, C. Bouillier, D. Sitterlin, S. Blouquit-Laye, M. Galloux, C. Zimmer, J.-F. Eleouet, M.-A. Rameix-Welti, Functional organization of cytoplasmic inclusion bodies in cells infected by respiratory syncytial virus. *Nat. Commun.* **8**, 563 (2017).

6. M. G. Rudolph, I. Kraus, A. Dickmanns, M. Eickmann, W. Garten, R. Ficner, Crystal structure of the borna disease virus nucleoprotein. *Structure* **11**, 1219–1226 (2003).

7. T. J. Green, X. Zhang, G. W. Wertz, M. Luo, Structure of the vesicular stomatitis virus nucleoprotein-RNA complex. *Science* **313**, 357–360 (2006).

8. A. A. V. Albertini, A. K. Wernimont, T. Muziol, R. B. G. Ravelli, C. R. Clapier, G. Schoehn, W. Weissenhorn, R. W. H. Ruigrok, Crystal structure of the rabies virus nucleoprotein-RNA complex. *Science* **313**, 360–363 (2006).

9. T. J. Green, M. Luo, Structure of the vesicular stomatitis virus nucleocapsid in complex with the nucleocapsid-binding domain of the small polymerase cofactor, P. *Proc. Natl. Acad. Sci. U.S.A.* **106**, 11713–11718 (2009).

10. R. G. Tawar, S. Duquerroy, C. Vonrhein, P. F. Varela, L. Damier-Piolle, N. Castagné, K. MacLellan, H. Bedouelle, G. Bricogne, D. Bhella, J.-F. Eléouët, F. A. Rey, Crystal structure of a nucleocapsid-like nucleoprotein-RNA complex of respiratory syncytial virus. *Science* **326**, 1279–1283 (2009).

11. I. Gutsche, A. Desfosses, G. Effantin, W. L. Ling, M. Haupt, R. W. H. Ruigrok, C. Sachse, G. Schoehn, Near-atomic cryo-EM structure of the helical measles virus nucleocapsid. *Science* **348**, 704–707 (2015).

12. S. G. Guryanov, L. Liljeroos, P. Kasaragod, T. Kajander, S. J. Butcher, Crystal structure of the measles virus nucleoprotein core in complex with an N-terminal region of phosphoprotein. *J. Virol.* **90**, 2849–2857 (2015).

13. R. N. Kirchdoerfer, D. M. Abelson, S. Li, M. R. Wood, E. O. Saphire, Assembly of the Ebola virus nucleoprotein from a chaperoned VP35 complex. *Cell Rep.* **12**, 140–149 (2015).

14. D. W. Leung, D. Borek, P. Luthra, J. M. Binning, M. Anantpadma, G. Liu, I. B. Harvey, Z. Su, A. Endlich-Frazier, J. Pan, R. S. Shabman, W. Chiu, R. A. Davey, Z. Otwinowski, C. F. Basler, G. K. Amarasinghe, An intrinsically disordered peptide from Ebola virus VP35 controls viral RNA synthesis by modulating nucleoprotein-RNA interactions. *Cell Rep.* **11**, 376–389 (2015).

15. M. Renner, M. Bertinelli, C. Leyrat, G. C. Paesen, L. F. Saraiva de Oliveira, J. T. Huiskonen, J. M. Grimes, Nucleocapsid assembly in pneumoviruses is regulated by conformational switching of the N protein. *eLife* **5**, e12627 (2016).

16. Y. Sugita, H. Matsunami, Y. Kawaoka, T. Noda, M. Wolf, Cryo-EM structure of the Ebola virus nucleoprotein-RNA complex at 3.6 Å resolution. *Nature* **563**, 137–140 (2018).

17. F. C. A. Gérard, J.-M. Bourhis, C. Mas, A. Branchard, D. D. Vu, S. Varhoshkova, C. Leyrat, M. Jamin, Structure and dynamics of the unassembled nucleoprotein of rabies virus in complex with its phosphoprotein chaperone module. *Viruses* **14**, 2813 (2022).

18. R. N. Kirchdoerfer, E. O. Saphire, A. B. Ward, Cryo-EM structure of the Ebola virus nucleoprotein–RNA complex. *Acta Crystallogr. F Struct. Biol. Commun.* **75**, 340–347 (2019).

19. L. Zinzula, F. Beck, S. Klumpe, S. Bohn, G. Pfeifer, D. Bollschweiler, I. Nagy, J. M. Plitzko, W. Baumeister, Cryo-EM structure of the cetacean morbillivirus nucleoprotein-RNA complex. *J. Struct. Biol.* **213**, 107750 (2021).

20. Y. Fujita-Fujiharu, Y. Sugita, Y. Takamatsu, K. Houri, M. Igarashi, Y. Muramoto, M. Nakano, Y. Tsunoda, I. Taniguchi, S. Becker, T. Noda, Structural insight into Marburg virus nucleoprotein–RNA complex formation. *Nat. Commun.* **13**, 1191 (2022).

21. S. Hu, Y. Fujita-Fujiharu, Y. Sugita, L. Wendt, Y. Muramoto, M. Nakano, T. Hoenen, T. Noda, Cryoelectron microscopic structure of the nucleoprotein–RNA complex of the European filovirus, Lloviu virus. *PNAS Nexus* **2**, pgad120 (2023).

22. L. Zinzula, F. Beck, M. Camasta, S. Bohn, C. Liu, D. Morado, A. Bracher, J. M. Plitzko, W. Baumeister, Cryo-EM structure of single-layered nucleoprotein-RNA complex from Marburg virus. *Nat. Commun.* **15**, 10307 (2024).

23. X. Song, H. Shan, Y. Zhu, S. Hu, L. Xue, Y. Chen, W. Ding, T. Niu, J. Gu, S. Ouyang, Q.-T. Shen, Z.-J. Liu, Self-capping of nucleoprotein filaments protects the Newcastle disease virus genome. *eLife* **8**, e45057 (2019).

24. D.-S. Ker, H. T. Jenkins, S. J. Greive, A. A. Antson, CryoEM structure of the Nipah virus nucleocapsid assembly. *PLOS Pathog.* **17**, e1009740 (2021).

25. L. Gonnin, A. Desfosses, M. Bacia-Verloop, D. Chevret, M. Galloux, J.-F. Éléouët, I. Gutsche, Structural landscape of the respiratory syncytial virus nucleocapsids. *Nat. Commun.* **14**, 5732 (2023).

26. H. H. Niller, K. Angstwurm, D. Rubbenstroth, K. Schlottau, A. Ebinger, S. Giese, S. Wunderlich, B. Banas, L. F. Forth, D. Hoffmann, D. Höper, M. Schwemmle, D. Tappe, J. Schmidt-Chanasit, D. Nobach, C. Herden, C. Brochhausen, N. Velez-Char, A. Mamilos, K. Utpatel, M. Evert, S. Zoubaa, M. J. Riemenschneider, V. Ruf, J. Herms, G. Rieder, M. Errath, K. Matiasek, J. Schlegel, F. Liesche-Starnecker, B. Neumann, K. Fuchs, R. A. Linker, B. Salzberger, T. Freilinger, L. Gartner, J. J. Wenzel, U. Reischl, W. Jilg, A. Gessner, J. Jantsch, M. Beer, B. Schmidt, Zoonotic spillover infections with Borna disease virus 1 leading to fatal human encephalitis, 1999–2019: An epidemiological investigation. *Lancet Infect. Dis.* **20**, 467–477 (2020).

27. Y. Matsumoto, Y. Hayashi, H. Omori, T. Honda, T. Daito, M. Horie, K. Ikuta, K. Fujino, S. Nakamura, U. Schneider, G. Chase, T. Yoshimori, M. Schwemmle, K. Tomonaga, Bornavirus closely associates and segregates with host chromosomes to ensure persistent intranuclear infection. *Cell Host Microbe* **11**, 492–503 (2012).

28. M. Hock, I. Kraus, G. Schoehn, M. Jamin, C. Andrei-Selmer, W. Garten, W. Weissenhorn, RNA induced polymerization of the Borna disease virus nucleoprotein. *Virology* **397**, 64–72 (2010).

29. K. Jamali, L. Käll, R. Zhang, A. Brown, D. Kimanius, S. H. W. Scheres, Automated model building and protein identification in cryo-EM maps. *Nature* **628**, 450–457 (2024).

30. M. Alayyoubi, G. P. Leser, C. A. Kors, R. A. Lamb, Structure of the paramyxovirus parainfluenza virus 5 nucleoprotein–RNA complex. *Proc. Natl. Acad. Sci. U.S.A.* **112**, E1792–E1799 (2015).

31. H. Shan, X. Su, T. Li, Y. Qin, N. Zhang, L. Yang, L. Ma, Y. Bai, L. Qi, Y. Liu, Q.-T. Shen, Structural plasticity of mumps virus nucleocapsids with cryo-EM structures. *Commun. Biol.* **4**, 833 (2021).

32. J. García, B. García-Barreno, A. Vivo, J. A. Melero, Cytoplasmic inclusions of respiratory syncytial virus-infected cells: Formation of inclusion bodies in transfected cells that coexpress the nucleoprotein, the phosphoprotein, and the 22K protein. *Virology* **195**, 243–247 (1993).

33. M. Chenik, K. Chebli, Y. Gaudin, D. Blondel, In vivo interaction of rabies virus phosphoprotein (P) and nucleoprotein (N): Existence of two N-binding sites on P protein. *J. Gen. Virol.* **75**, 2889–2896 (1994).

34. A. Derdowski, T. R. Peters, N. Glover, R. Qian, T. J. Utley, A. Burnett, J. V. Williams, P. Spearman, J. E. Crowe, Human metapneumovirus nucleoprotein and phosphoprotein interact and provide the minimal requirements for inclusion body formation. *J. Gen. Virol.* **89**, 2698–2708 (2008).

35. S. Zhang, L. Chen, G. Zhang, Q. Yan, X. Yang, B. Ding, Q. Tang, S. Sun, Z. Hu, M. Chen, An amino acid of human parainfluenza virus type 3 nucleoprotein is critical for template function and cytoplasmic inclusion body formation. *J. Virol.* **87**, 12457–12470 (2013).

36. Y. Zhou, J. M. Su, C. E. Samuel, D. Ma, Measles virus forms inclusion bodies with properties of liquid organelles. *J. Virol.* **93**, e00948–19 (2019).

37. Y. Hirai, K. Tomonaga, M. Horie, Borna disease virus phosphoprotein triggers the organization of viral inclusion bodies by liquid-liquid phase separation. *Int. J. Biol. Macromol.* **192**, 55–63 (2021).

38. P. Ge, J. Tsao, S. Schein, T. J. Green, M. Luo, Z. H. Zhou, Cryo-EM model of the bullet-shaped vesicular stomatitis virus. *Science* **327**, 689–693 (2010).

39. K. Zhou, Z. Si, P. Ge, J. Tsao, M. Luo, Z. H. Zhou, Atomic model of vesicular stomatitis virus and mechanism of assembly. *Nat. Commun.* **13**, 5980 (2022).

40. S. Jenni, J. A. Horwitz, L.-M. Bloyet, S. P. J. Whelan, S. C. Harrison, Visualizing molecular interactions that determine assembly of a bullet-shaped vesicular stomatitis virus particle. *Nat. Commun.* **13**, 4802 (2022).

41. C. Riedel, D. Vasishtan, V. Pražák, A. Ghanem, K.-K. Conzelmann, T. Rümenapf, Cryo EM structure of the rabies virus ribonucleoprotein complex. *Sci. Rep.* **9**, 9639 (2019).

42. M. J. Conley, J. M. Short, A. M. Burns, J. Streetley, J. Hutchings, S. E. Bakker, B. J. Power, H. Jaffery, J. Haney, G. Zanetti, P. R. Murcia, M. Stewart, R. Fearns, S. Vijayakrishnan, D. Bhella, Helical ordering of envelope-associated proteins and glycoproteins in respiratory syncytial virus. *EMBO J.* **41**, e109728 (2022).

43. G. Pauli, H. Ludwig, Increase of virus yields and releases of Borna disease virus from persistently infected cells. *Virus Res.* **2**, 29–33 (1985).

44. T. Noda, L. Kolesnikova, L. Kolesnikova, S. Becker, S. Becker, Y. Kawaoka, Y. Kawaoka, The importance of the NP: VP35 ratio in Ebola virus nucleocapsid formation. *J Infect Dis* **204**, S878–S883 (2011).

45. F. Chenavier, L. F. Estrozi, J.-M. Teulon, E. Zarkadas, L.-L. Freslon, J.-L. Pellequer, R. W. H. Ruigrok, G. Schoehn, A. Ballandras-Colas, T. Crépin, Cryo-EM structure of influenza helical nucleocapsid reveals NP-NP and NP-RNA interactions as a model for the genome encapsidation. *Sci. Adv.* **9**, eadj9974 (2023).

46. F. Chenavier, E. Zarkadas, L.-L. Freslon, A. J. Stelfox, G. Schoehn, R. W. H. Ruigrok, A. Ballandras-Colas, T. Crépin, Influenza a virus antiparallel helical nucleocapsid-like pseudo-atomic structure. *Nucleic Acids Res.* **53**, gkae1211 (2025).

47. R. Peng, X. Xu, B. Nepal, Y. Gong, F. Li, M. B. Ferretti, M. Zhou, K. W. Lynch, G. M. Burslem, S. Kortagere, R. Marmorstein, Y.-W. Chang, Molecular basis of influenza ribonucleoprotein complex assembly and processive RNA synthesis. *Science* **388**, eadq7597 (2025).

48. Y.-S. Tang, S. Xu, Y.-W. Chen, J.-H. Wang, P.-C. Shaw, Crystal structures of influenza nucleoprotein complexed with nucleic acid provide insights into the mechanism of RNA interaction. *Nucleic Acids Res.* **49**, 4144–4154 (2021).

49. H. Kang, Y. Yang, Y. Liu, M. Li, L. Zhang, Y. Lin, L. Witte, K.-Y. Chen, W. Song, Z. Xu, X. He, L. W. Guddat, Y. Guo, L. Yan, Y. Gao, E. Fodor, Z. Rao, Z. Lou, Coupling of polymerase-nucleoprotein-RNA in an influenza virus mini ribonucleoprotein complex. *Nat. Commun.* **16**, 9741 (2025).

50. F. Iseni, R. W. Ruigrok, D. Blondel, A. Barge, F. Baudin, Characterization of rabies virus nucleocapsids and recombinant nucleocapsid-like structures. *J. Gen. Virol.* **79**, 2909–2919 (1998).

51. C. Esneau, B. Raynal, P. Roblin, S. Brûlé, C.-A. Richard, J. Fix, J.-F. Eléouët, M. Galloux, Biochemical characterization of the respiratory syncytial virus N0-P complex in solution. *J. Biol. Chem.* **294**, 3647–3660 (2019).

52. B. Liu, S. Dong, G. Li, W. Wang, X. Liu, Y. Wang, C. Yang, Z. Rao, Y. Guo, Structural insight into nucleoprotein conformation change chaperoned by VP35 peptide in Marburg virus. *J. Virol.* **91**, e00825–17 (2017).

53. D. N. Mastronarde, Automated electron microscope tomography using robust prediction of specimen movements. *J. Struct. Biol.* **152**, 36–51 (2005).

54. J. Zivanov, T. Nakane, B. O. Forsberg, D. Kimanius, W. J. H. Hagen, E. Lindahl, S. H. W. Scheres, New tools for automated high-resolution cryo-EM structure determination in RELION-3. *eLife* **7**, e42166 (2018).

55. A. Punjani, J. L. Rubinstein, D. J. Fleet, M. A. Brubaker, cryoSPARC: Algorithms for rapid unsupervised cryo-EM structure determination. *Nat. Methods* **14**, 290–296 (2017).

56. A. Punjani, H. Zhang, D. J. Fleet, Non-uniform refinement: Adaptive regularization improves single-particle cryo-EM reconstruction. *Nat. Methods* **17**, 1214–1221 (2020).

57. T. Bepler, A. Morin, M. Rapp, J. Brasch, L. Shapiro, A. J. Noble, B. Berger, TOPAZ: A positive-unlabeled convolutional neural network CryoEM particle picker that can pick any size and shape particle. *Microsc. Microanal.* **25**, 986–987 (2019).

58. T. D. Goddard, C. C. Huang, E. C. Meng, E. F. Pettersen, G. S. Couch, J. H. Morris, T. E. Ferrin, UCSF ChimeraX: Meeting modern challenges in visualization and analysis. *Protein Sci.* **27**, 14–25 (2018).

59. P. Emsley, B. Lohkamp, W. G. Scott, K. Cowtan, Features and development of Coot. *Acta Crystallogr. D Biol. Crystallogr.* **66**, 486–501 (2010).

60. G. N. Murshudov, P. Skubák, A. A. Lebedev, N. S. Pannu, R. A. Steiner, R. A. Nicholls, M. D. Winn, F. Long, A. A. Vagin, *REFMAC* 5 for the refinement of macromolecular crystal structures. *Acta Crystallogr. D Biol. Crystallogr.* **67**, 355–367 (2011).

61. K. Yamashita, C. M. Palmer, T. Burnley, G. N. Murshudov, Cryo-EM single-particle structure refinement and map calculation using *Servalcat*. *Acta Crystallogr. D Struct. Biol.* **77**, 1282–1291 (2021).

62. R. A. Nicholls, M. Fischer, S. McNicholas, G. N. Murshudov, Conformation-independent structural comparison of macromolecules with *ProSMART*. *Acta Crystallogr. D Biol. Crystallogr.* **70**, 2487–2499 (2014).

63. A. Brown, F. Long, R. A. Nicholls, J. Toots, P. Emsley, G. Murshudov, Tools for macromolecular model building and refinement into electron cryo-microscopy reconstructions. *Acta Crystallogr. D Biol. Crystallogr.* **71**, 136–153 (2015).

64. A. Reuter, M. Horie, D. Höper, A. Ohnemus, A. Narr, M. Rinder, M. Beer, P. Staeheli, D. Rubbenstroth, Synergistic antiviral activity of ribavirin and interferon-α against parrot bornaviruses in avian cells. *J. Gen. Virol.* **97**, 2096–2103 (2016).

65. T. Williams, C. Kelley, Gnuplot: An Interactive Plotting Program, version 6.0 (2024); http://gnuplot.sourceforge.net/.

66. K. Katoh, D. M. Standley, MAFFT multiple sequence alignment software version 7: Improvements in performance and usability. *Mol. Biol. Evol.* **30**, 772–780 (2013).

67. X. Robert, P. Gouet, Deciphering key features in protein structures with the new ENDscript server. *Nucleic Acids Res.* **42**, W320–W324 (2014).

68. M. Aggarwal, G. P. Leser, C. A. Kors, R. A. Lamb, Structure of the paramyxovirus parainfluenza virus 5 nucleoprotein in complex with an amino-terminal peptide of the phosphoprotein. *J. Virol.* **92**, e01304-17 (2018).

69. F. Yabukarski, P. Lawrence, N. Tarbouriech, J.-M. Bourhis, E. Delaforge, M. R. Jensen, R. W. H. Ruigrok, M. Blackledge, V. Volchkov, M. Jamin, Structure of Nipah virus unassembled nucleoprotein in complex with its viral chaperone. *Nat. Struct. Mol. Biol.* **21**, 754–759 (2014).

70. C. Leyrat, F. Yabukarski, N. Tarbouriech, E. A. Ribeiro, M. R. Jensen, M. Blackledge, R. W. H. Ruigrok, M. Jamin, Structure of the vesicular stomatitis virus N^0-P complex. *PLOS Pathog.* **7**, e1002248 (2011).

71. T. I. Croll, *ISOLDE* : A physically realistic environment for model building into low-resolution electron-density maps. *Acta Crystallogr. D Struct. Biol.* **74**, 519–530 (2018).
